# Potential reasons for the decline of new HIV cases among people who inject drugs (PWID) in Kyrgyzstan

**DOI:** 10.1371/journal.pone.0348970

**Published:** 2026-05-26

**Authors:** Ainura Moldokmatova, Wirichada Pan-Ngum, Proochista Ariana, Caroline Franco, Ricardo Aguas, Chynarkul Zhumalieva, Zhursunai Asangozhoeva, Lisa White

**Affiliations:** 1 Nuffield Department of Medicine, University of Oxford, United Kingdom; 2 Mahidol-Oxford Tropical Medicine Research Unit (MORU), Mahidol University, Bangkok, Thailand; 3 Biostatistics and Health Data Science, Institute of Applied Health Sciences, School of Medicine, Medical Sciences and Nutrition, University of Aberdeen, United Kingdom; 4 Republican Centre for Bloodborne Viral Hepatitis and HIV Control, Bishkek, Kyrgyzstan; 5 Biology Department, University of Oxford, United Kingdom; Texas A&M University College Station, UNITED STATES OF AMERICA

## Abstract

There has been a declining trend in the number of reported cases of human immunodeficiency virus (HIV) among people who inject drugs (PWID) in Kyrgyzstan. The local HIV and public health community has suggested that this decline may be driven by interventions targeting HIV transmission among PWID, changes in drug use patterns, or possible underreporting of new HIV cases. The present study aims to examine these hypotheses using a deterministic compartmental model. Three intervention scenarios were evaluated to assess their impact on PWID population trends, HIV incidence, and case reporting. Scenario I (baseline) included needle and syringe exchange programmes, opioid substitution therapy, behavioural interventions, and pre-exposure prophylaxis, collectively referred to as preventive interventions, alongside antiretroviral therapy (ART). Scenario II excluded preventive interventions while maintaining ART. Scenario III excluded both preventive interventions and ART. The model results suggest that the decline in PWID may be a key factor contributing to the reduction of the HIV epidemic in this group. While preventive interventions and ART are unlikely to have been the primary drivers of HIV incidence trends, they appear to have played a meaningful role in reducing the overall HIV burden. Furthermore, the model indicates that observed trends in reported HIV incidence are likely to reflect changes in testing behaviour rather than actual fluctuations in the number of new infections.

## Introduction

Kyrgyzstan’s geographic position along the northern drug trafficking route from Afghanistan has facilitated easy access to heroin and other opiates, significantly affecting the population of people who inject drugs (PWID) and contributing to the HIV epidemic. In the early stages of the epidemic, PWID accounted for 94 per cent of newly reported HIV cases. By 2022, the number of newly reported HIV cases among PWID had declined by 92 per cent [1].

The country reports that the decline in the HIV epidemic among PWID may be attributed to the effective implementation of targeted interventions [2,3] such as needle and syringe programs (NSP), opioid substitution therapy (OST) with methadone, behavioural interventions involving the PWID community, and various other harm reduction measures aimed at reducing risky behaviours and improving health-seeking practices. Additionally, the country has recently initiated pre-exposure prophylaxis (PrEP) services for this population. However, some national experts hypothesise that a proportion of individuals who inject drugs may be transitioning to alternative, potentially non-injecting substances [4]. Although empirical evidence documenting such a shift remains limited, it cannot be excluded as a possible contributing factor to the observed decline in HIV. At the same time, surveillance studies in Kyrgyzstan have observed shifts in the PWID population structure, with a declining proportion of younger individuals and an increasing proportion of older age groups, suggesting a possible ageing cohort of PWID in the country [5–7]. Notably, national estimation studies have consistently reported a stable population size of 25,000 individuals over time [8,9], although recent data from the National Narcology Centre indicate a decline in the number of registered PWID [10]. Another possibility proposed by experts is the potential underreporting of new HIV cases [11,12].

This study evaluates hypotheses concerning the impact of changes in population size, harm reduction interventions, and potential underreporting on the HIV epidemic in this population, using a mathematical transmission model that builds upon established frameworks developed by Li and Granich [13,14]. This represents a novel application of this approach in the Kyrgyz context. The findings provide valuable insights into the impact of preventative interventions and demographic shifts, both of which are subjects of ongoing discussion in national public health dialogues.

## Methods

The modelling process was conducted in several steps. First, we calibrated the model using an optimisation approach against reported new HIV cases among PWID, assuming time-varying annual coverage levels for HIV testing. This gave us the value of the unknown parameters: transmission coefficient β(), transition from the antiretroviral treatment (ART) to non-treatment state ω() and entry rate to the PWID population (μ). It should be noted that other model outputs (the number of HIV deaths and registered PWID) were visually compared with the observed data to further validate the model. We then assessed the impact of different intervention scenarios and tested the following hypotheses by using scenario-based analysis approach.

***Hypothesis I:***The PWID population has declined from 1984 to 2024

***Hypothesis II:***The interventions targeted at HIV prevention and improvement of ART outcomes were effective

***Hypothesis III:***HIV reported incidence tracks changes in testing rate

The scripts of the model are publicly available at: https://github.com/amoldokmatova/HIV-Restrospective.

### Data sources

Model parameters were informed by peer-reviewed publications and, where possible, publicly available epidemiological data. These include data on HIV transmission risks across different disease stages, HIV progression with and without ART, stage durations, and AIDS-related mortality. Additionally, yearly HIV testing coverage data, both active and passive, based on diagnostic algorithms, linkage to antiretroviral therapy (accounting for disease stage and updates in national guidelines), and historical coverage of harm reduction and related services, considering their demonstrated effectiveness, were incorporated. Where data gaps existed, expert opinion was sought through personal contacts and networks. Comprehensive information on parameter values and their corresponding sources is presented in S1 File, except for the yearly HIV testing coverage and annual AIDS-related deaths data, which are available upon request from the Republican Centre for Bloodborne Viral Hepatitis and HIV Control, Kyrgyzstan. The values on the HIV transmission coefficient and ART adherence were estimated due to the lack of available information.

Additionally, we accessed depersonalised data from the Kyrgyz Sentinel Surveillance on HIV among PWID for the years 2006–2010 during the period of February 20–25, 2025, to further examine the population structure of PWID. The accessed data were limited to information on population structure.

### Model framework

**Fig 1:**
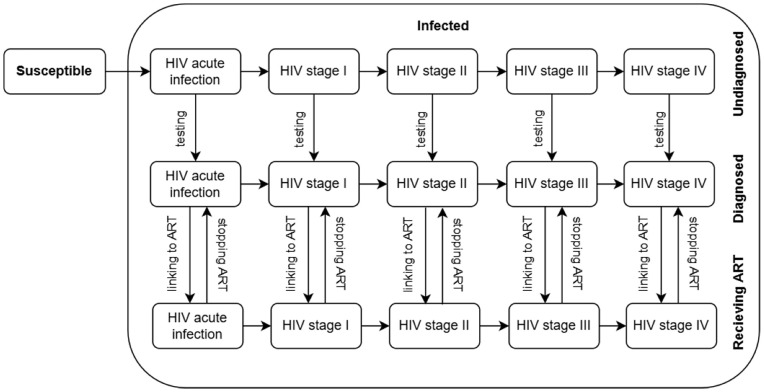
HIV transmission and disease stage progression. The PWID population is divided into two main compartments: susceptible to and infected with HIV. The infected compartment is further subdivided according to different stages of HIV progression. Those infected are categorised into two groups: undiagnosed and diagnosed. The movement from diagnosed to undiagnosed occurs at any HIV progression stage through testing (passive or active). ART sub-compartments are integrated within the infected population. Individuals can initiate or discontinue ART at any stage following diagnosis. Disease progression among those receiving ART is slower compared to those not on treatment.

A deterministic compartmental mathematical model was developed to conduct scenario analyses assessing the potential impact of preventative interventions and ART on HIV transmission and mortality among PWID. The model is an adapted version of previously published frameworks by Li and Granich [13,14], modified to reflect the epidemiological and service delivery context in Kyrgyzstan. It consists of susceptible and infected compartments, with the latter structured by HIV disease stages, beginning with acute infection and progressing through stages one to four (AIDS as a final stage). Each stage is further stratified by diagnosis and treatment status (undiagnosed, diagnosed, and receiving ART). The detailed description of the model structure is provided below, with the full model framework and equations available in S2 File, Fig 1.

As shown in Fig 1, it is assumed that an individual, once infected, progresses through the course of the disease, which includes the acute infection phase followed by clinical stages one through four, as defined by the World Health Organisation (WHO) [15,16]. The definitions of HIV stages are provided in S2 File Box 1.

At any stage of infection, an individual may undergo HIV testing through either active testing, where healthcare providers approach the community to offer HIV screening services, or passive testing, which occurs when an individual, having developed symptoms, seeks medical attention and is diagnosed with HIV. Alternatively, the individual may remain undiagnosed until reaching the final stage, AIDS (Fig 1).

HIV diagnosis is confirmed after an individual receives at least three positive test results, in accordance with the HIV testing algorithms outlined in the national clinical protocol [17]. HIV detection sensitivity is lower during the acute infection phase, although the risk of transmission is highest during this phase and is comparatively higher in the final stages than in the asymptomatic stages (S1 Tables A-B in S1 File).

Individuals diagnosed with HIV may begin ART, typically based on the stage of their illness (Fig 1), and the model reflects this by incorporating policy changes related to ART eligibility by HIV stage (S1 Table C in S1 File). Initially, only individuals with severe immunosuppression (CD4 ≤ 200 cells/ml) and/or those with AIDS-related severe conditions (stage IV) were eligible for treatment [18,19]. However, with the release of new WHO guidelines and increased drug availability, eligibility was expanded to individuals with lower immunosuppression levels: in 2010, those with CD4 < 350 cells/ml and/or advanced symptoms (stage III) [20]; in 2013, those with CD4 < 500 cells/ml and/or with mild symptoms (stage II) [21] and in 2017, the ‘Test and Treat’ approach was implemented, allowing treatment for all individuals diagnosed with HIV [22,23].

Additionally, the model assumes that a certain proportion of individuals will receive treatment immediately following their diagnosis. Adherence to treatment is estimated and assumed to be consistent across all disease stages, without distinguishing between short- and long-term adherence.

### Preventative interventions

Evidence indicates that harm reduction programs, including NSP [24–27], OST [28–30] and behavioural change interventions [31–33], are effective in reducing HIV transmission and promoting health-seeking behaviour among PWID. Evidence on the effectiveness of PrEP among PWID is limited to a randomised controlled trial in Bangkok, which demonstrated a 48 per cent reduction in HIV incidence within this population [34].

The above interventions are incorporated into the model, reflecting the historical coverage levels for each intervention in Kyrgyzstan (S1 Tables D-G in S1 File). According to national reports, NSP and behavioural interventions have been relatively successful, reaching a significant proportion of PWID [35]. In contrast, the OST program faced implementation challenges and was able to reach only 4.4 per cent of the target population in 2023 [36]. The PrEP is a relatively new intervention in Kyrgyzstan, launched in 2019; therefore, it is too early to assess its full impact, although it is still included in the model’s scenario analysis.

It is assumed that all four interventions impact the transmission coefficient (β), while OST and behavioural interventions influence ART linkage rates (α), the proportion of individuals starting ART after diagnosis (φ), and ART non-adherence rates, i.e., moving rate from ART to non-ART state (ω). Further details are provided in S1 Table H in S1 File.

### Model fitting and validation

**Fig 2:**
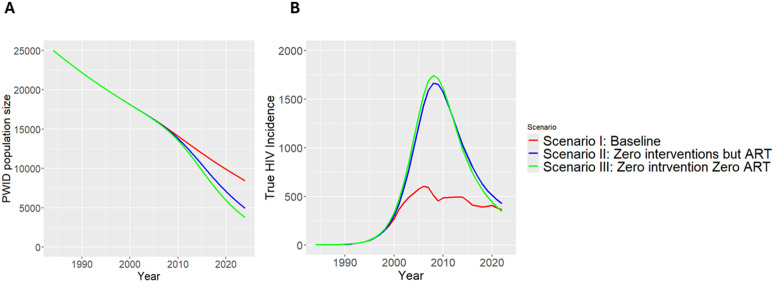
Estimated annual PWID population (A) and yearly true HIV incidence, including both reported and unreported (B), by intervention scenarios. The red curve illustrates the baseline intervention scenario (Scenario I), showing the projected trend in the PWID population (Panel A) and HIV incidence (Panel B) under existing interventions. The blue curve (Scenario II) depicts a hypothetical situation in which all interventions, NSP, OST, PrEP, and behavioural, are removed retrospectively, while ART is retained. The green curve (Scenario III) shows the impact of removing both ART and all other interventions.

The model was fitted using available data on reported annual HIV cases among PWID for the period 2002–2023, employing an optimisation approach to minimise the negative log-likelihood (NLL). The likelihood function was based on the Poisson distribution, with the model’s estimated HIV incidence representing the average annual rate of new infections. This calibration approach allowed for the estimation of the transmission coefficient (β) and the transition rate from the ART state to the non-ART state (ω). To enhance robustness, the model was fitted across a range of PWID population replenishment scenarios, in which population entry rates (μ) were set equal to, lower than, or higher than the population exit rates (κ), thereby allowing for exploration of both stable and dynamic population size assumptions (S2 Fig 2a in S2 File).

In addition, the Akaike Information Criterion (AIC) was applied for model selection [37], with AIC defined as $AIC={2n}_{p}+2NLL$, where $n_{p}$ denotes the number of estimated parameters and NLL is the negative log-likelihood value. Models with an AIC difference from the best-performing model **(**$\Delta {AIC}_{\iota }={AIC}_{\iota }-{AIC}_{min}$**)** of four or more are considered to have substantially less support (see **S2 Appendix, Table A** for more details on ${AIC}_{\iota }$-based model selection). Using this criterion, the population replenishment scenarios providing a good fit were found to range from μ ***=*** κ ***− 0.035*** to μ ***=*** κ ***− 0.005***.

Additional validation using annual HIV-attributable death data from the National AIDS Centre indicated that modelled deaths were underestimated when (κ *− 0.015) ≥* μ and overestimated for μ *≤ (*κ *− 0.035).* Validation against the number of registered PWID, as reported by the National Narcology Centre, showed an exact fit for μ *≤ (*κ *− 0.035),* suggesting underestimation of the actual population due to social, cultural, and legal factors that discourage some PWID from registering at drug treatment facilities (S2 Fig 2b in S2 File). Following the comparison of these two additional model outputs with the observed data, the range (κ *− 0.030) ≤* μ *≤ (*κ *− 0.020)* was identified as being consistent with both the annual number of HIV-attributable deaths and the number of registered PWID. Within this range, the scenario with μ *=* κ *− 0.020* yielded the lowest objective function value (OFV = 169.17) and was selected for subsequent hypothesis testing related to the observed decline in reported HIV cases among PWID in Kyrgyzstan.

### Sensitivity Analysis: Effect of uncertainty levels of other input parameters on the HIV incidence and AIDS-attributable mortality

**Fig 3:**
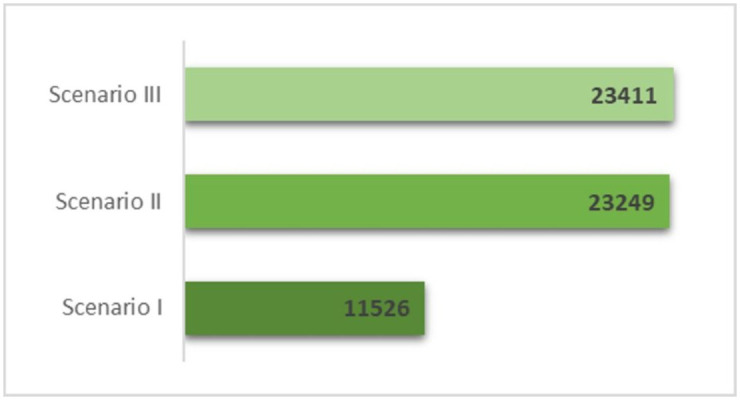
Cumulative true HIV incidence (including both reported and unreported) during the model simulation period (1984–2024) across intervention scenarios. Each column represents a different scenario: Scenario I (baseline), Scenario II (preventative interventions removed, ART retained), and Scenario III (both preventative interventions and ART removed).

We conducted a deterministic global sensitivity analysis to evaluate how uncertainty in multiple model parameters, including yearly HIV screening coverage, confirmatory testing proportions, population entry and exit rates, the transmission coefficient, and transitions from ART to non-ART status, affects model outcomes (HIV incidence and AIDS-attributable mortality). Parameters were varied systematically across predefined ranges using a full-factorial grid sampling design, with three evenly spaced levels per parameter. All possible combinations of parameter levels were evaluated. The grid sampling generated uniformly spaced values within each parameter range (lower bound, midpoint, and upper bound). Results were summarized by examining outcome distributions across levels of individual parameters, thereby estimating their marginal influence within the jointly varied parameter space (S2 Fig 3 in S2 File).

The sensitivity analysis indicated that the maximum yearly HIV incidence under baseline PrEP, OST, NSP, and behavioural interventions was most sensitive to the PWID population exit rate, followed by the population entry rate and the transmission coefficient. In contrast, the outcome showed minimal sensitivity to yearly HIV screening coverage and the proportion of individuals proceeding to confirmatory testing. Moderate sensitivity was observed for the proportions transitioning from initial screening to the second test and subsequently to the final confirmatory test for HIV diagnosis. Cumulative HIV incidence and cumulative AIDS-attributable mortality exhibited patterns similar to those observed for maximum yearly HIV incidence. Both outcomes were highly sensitive to the population exit rate, followed by the population entry rate and the transmission coefficient, and demonstrated comparatively lower sensitivity to HIV screening coverage, confirmatory testing parameters, and transitions from ART to non-ART status.

## Results

Intervention scenario analysis is used to evaluate the hypotheses mentioned above. Each intervention scenario, as outlined in Box 1, is assessed for the population replenishment scenarios with the entry rate (μ) less than the exit rate (κ) by the value of 0.020 (μ *=* κ *− 0.020*).

Box 1. Model intervention scenarios for the population replenishment of μ = κ − 0.020

Scenario I: Baseline (with NSP, OST, PrEP and behavioural interventions and ART)

Scenario II: Removing NSP, OST, PrEP and behavioural interventions only, and keeping ART

Scenario III: Removing NSP, OST, PrEP and behavioural interventions and ART

### Hypothesis I: Has the PWID population decreased between 1984 and 2024?

As outlined in the model fitting section, among the population replenishment scenarios representing declining, stable, and increasing populations, the scenario with reduced population influx provided the best fit to the observed data (S2 Table A in S1 File). The model-predicted PWID population size over time under each intervention scenario is shown in Fig 2A.

**Fig 4:**
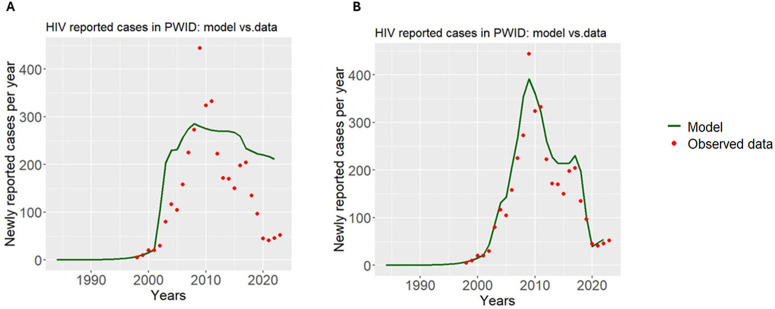
The modelled reported HIV incidence using (A) stable testing rates and (B) time-varying testing rates. The green curve represents the model output, while the red dots indicate the actual reported HIV incidence.

Consistent with the model outcomes, analyses of the working Sentinel Surveillance datasets (Republican AIDS Centre, 2006–2010) among PWID revealed a declining trend in HIV prevalence among younger age groups and an increasing trend among older age groups over time (Fig 4 in Appendix S2).

### Hypothesis II: Were interventions targeted at HIV prevention and improvement of ART outcomes effective?

To test this hypothesis, the parameters for preventative interventions and ART were set to reflect either actual historical coverage levels (baseline), the removal of preventative interventions (Scenario II), or the removal of both preventative interventions and ART (Scenario III), while all other parameters were kept fixed. The outcomes of this analysis are interpreted in the context of a declining PWID population, as estimated during the testing of the previous hypothesis. As shown in Fig 2B, incidence would continue to decline even in the absence of ART and preventative interventions (Scenarios II and III). However, in the baseline scenario, the epidemic’s peak has likely been flattened.

Notably, the gap in cumulative HIV incidence between Scenarios II and III is minimal (162 infections), while the difference between the baseline scenario and Scenario II is substantial (11,723 infections) (Fig 3). This suggests that preventative interventions likely play a critical role in reducing the HIV burden among PWID in Kyrgyzstan, although they may not be the primary drivers of the epidemic’s overall trajectory.

### Hypothesis III: Does the reported incidence of HIV track changes in testing rates?

To test the hypothesis that reported HIV incidence tracks changes in testing rates, we first assumed a fixed annual screening coverage of 30 per cent of the estimated population, representing a high-coverage testing scenario in which reported diagnoses more closely reflect underlying HIV incidence. We then conducted two simulations comparing model-predicted HIV diagnoses under fixed versus time-varying testing rates. As shown in Figs 4A–B, the fixed screening scenario failed to reproduce the observed trends in HIV diagnoses. This suggests that temporal changes in testing rates contributed substantially to the reported incidence patterns.

**Fig 5:**
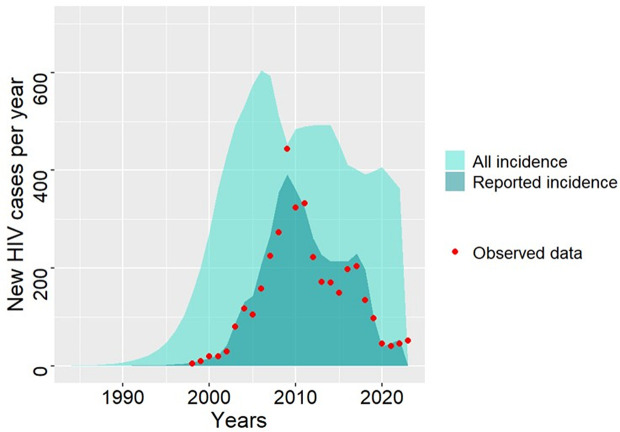
Yearly reported and unreported new HIV infection cases. The light turquoise polygon illustrates the estimated true incidence (reported and unreported), the dark turquoise polygon represents the reported incidence, and red dots indicate the actual reported HIV case data.

It should be noted that there appears to be a persistent difference between reported and unreported HIV cases over the course of the epidemic, indicating that some HIV-positive PWID may still be unaware of their infection (Fig 5).

## Discussion

The model findings align with potential shifts in the population of PWID, as suggested by proxy data. Thus, sentinel surveillance indicates a changing age distribution over time, specifically, a decline in the proportion of younger PWID (aged 20–29) and an increase in those aged 45 and older [5–7]. Given that the average age of first injection in Kyrgyzstan is approximately 20 years [38], this shift potentially reflects a reduction in the entry of new individuals into the PWID population. Supporting this assumption, the National Narcology Centre has documented a decline in the number of registered PWID in recent years [10]. Furthermore, considering the strong correlation between HIV and hepatitis C virus (HCV) transmission among PWID [39], the observed decline or stability in HCV prevalence, aside from Sokuluk village, may serve as an indirect indicator of reduced population replenishment [6,7]. However, more definitive insight would require HCV incidence data, which were unfortunately unavailable. Nevertheless, it is important to note that although the best-fitting scenarios suggest a declining PWID population, the model does not capture the mechanisms underlying this pattern.

The model’s estimation of the limited impact of preventative interventions on an already declining epidemic trajectory echoes McKeown’s argument that public health measures often contribute modestly to mortality declines already underway, with broader social determinants, such as improved nutrition, living conditions, and environmental factors, playing a more substantial role [40]. His historical analysis of England and Wales demonstrated that mortality rates began to decline before the widespread adoption of medical interventions, suggesting that such trends may have continued independently. A similar pattern may be emerging in Kyrgyzstan, where the observed decline in HIV incidence could reflect a shrinking population of PWID. However, while demographic shifts may potentially explain the decline, prevention and treatment interventions have likely played a key role in averting further infections and deaths, thereby mitigating the overall impact of the epidemic. This is supported by previous regional modelling studies, which have demonstrated the effectiveness of harm reduction strategies and ART in reducing HIV transmission among PWID. For example, projections by Cepeda and colleagues indicated that in Russian cities such as Omsk and Yekaterinburg, HIV prevalence would have increased significantly in the absence of such interventions. In contrast, scaling up OST, alongside NSP and expanded ART coverage, was shown to reduce new infections over time substantially [41].

Like all modelling studies, this work has several limitations. As mentioned earlier, it relies on various simplifying assumptions. Specifically, the model does not account for age- and gender-specific contact patterns, nor does it distinguish between transmission modes (sexual vs. non-sexual); instead, the transmission coefficient is estimated. Separating injecting-related and sexual transmission pathways in future model extensions would allow a more detailed assessment of how different interventions affect distinct routes of infection and could refine projections of long-term epidemic trends. Moreover, the model does not account for transmission dynamics between PWID and non-PWID populations, which may impact the overall accuracy of the epidemic’s spread. Model parameterisation presents another challenge encountered in this study. The model relies on various sources, including expert opinion, to aggregate historical coverage data for NSP, OST, behavioural interventions, PrEP, and community-based HIV screening, owing to the scattered and limited availability of data, particularly for the earlier periods of the epidemic. This challenge is common in many low- and middle-income countries and deserves consideration in strategic planning and national programmes.

## Conclusion

This study highlights several considerations for HIV policy in Kyrgyzstan. The findings point to the importance of maintaining and potentially adapting preventative interventions as part of the national HIV response. In light of possible changes in drug use patterns and the PWID population, it may be useful for policies to reflect evolving trends, drawing on ongoing data collection and analysis. Additionally, the study suggests the value of continued attention to HIV testing to help address potential challenges with reaching hidden PWID sub-populations.

## Supporting information

S1 File Model parameters. (DOCX)

S2 File Model framework, HIV stage definitions, Model fitting, Sensitivity analysis, Age groups distribution data. (DOCX)
