# Supplementary material for: Potential reasons for the decline of new HIV cases among people who inject drugs (PWID) in Kyrgyzstan
Source: PLoS One. 2026 May 26;21(5):e0348970. doi: 10.1371/journal.pone.0348970 (PMC13210176; doi:10.1371/journal.pone.0348970)
Supplement: S1 File — (DOCX) [file pone.0348970.s001.docx]

**S1.**

**Model parameters**

Note: The units for the parameter values are defined in years.

***Table A.*** Demographic, natural history of disease

| **Parameter** | | **Description** | **Value** | **Source** | **Comments** | **Fixed/**  **estimated** | |
| --- | --- | --- | --- | --- | --- | --- | --- |
|  |  | **Demographic: Entry/Leaving rates of people who inject drugs (PWID)** | | | | |  |
|  | $\mu$ | entry rate to PWID population |  |  | *Varies in population replenishment scenarios, where* $\mu$ *> or < or ==* $\kappa$ | Fixed |  |
|  | $\kappa$ | exit rate from PWID population | ***1/11*** | [1] | *Based on average duration of the injectable drug use* | Fixed |  |
|  | $\sigma$ | AIDS attributable death rate at stage IV (if not treated) | ***1.3*** *(9.2 months)* | [2]  [3] | *Proxy: Uganda (1990-2000)*  *Proxy: 7-11 months in Thailand, S.Africa, Uganda, Brazil (prior to 1998)* | Fixed  Fixed |  |
|  | $\upsilon$ | reduction of AIDS attributable death rate if on ART *(1=no decrease, 0=full decrease)* | ***0.5*** | [2] | *Proxy: in UK before HAART= 10 months, after= 19 months (refer to § 5 in Discussion)* | Fixed |  |
|  |  | **HIV transmission** | | | | |  |
|  | $\beta$ | transmission coefficient: number of contacts per year * prob of inf | ***-*** | *n/a* | No info/data | Estimated |  |
|  | $\gamma$ | reduced transmission if on ART *(1=no decrease, 0=full decrease)* | ***0.48 (9.6-72.2)*** | [4] | No data on ART’s effect on PWID. Based on proxy: RCT of PrEP in Bangkok | Fixed |  |
|  |  | Reduced/increased transmission if not on week 3-4 of acute infection stage | |  |  |  |  |
|  | $\iota_{a_{1}}$ | acute (wk 1) | ***5.5*** | [5] | *Acute (wk 1: ~690,000 c/ml)* | Fixed |  |
|  | $\iota_{a_{2}}$ | acute (wk 2) | ***1*** *(reference)* | [5] | *Acute (wk 2: ~125,000 c/ml)* | Fixed |  |
|  | $\iota_{a_{2}}$ | acute (wk 3-4) | ***0.4*** | [5] | *Acute (wk 3-4: ~50,000 c/ml)* | Fixed |  |
|  | $\iota_{1\_2}$ | stage I and II | ***0.1*** | [5] | *Stage 1-2 (~17,000 c/ml)* | Fixed |  |
|  | $\iota_{3}$ | stage III | ***0.3*** | [5] | *Stage 3 (~40,000 c/ml)* | Fixed |  |
|  | $\iota_{4}$ | stage IV | ***1.1*** | [5] | *Stage 4(~140,000 c/ml)* | Fixed |  |
|  |  | **HIV progression** | | | | |  |
|  |  | rate at which PLHIV progress through HIV stages if not treated | |  |  |  |  |
|  | $\theta_{a_{1}}$ | after the initial “eclipse phase”* to the week 2 of the acute infection stage | ***48*** *(1 week)* | [5], [6] |  | Fixed |  |
|  | $\theta_{a_{2}}$ | from the week 2 to week 3 of the acute infection | ***48*** *(1 week)* | [5] |  | Fixed |  |
|  | $\theta_{a_{3}}$ | from the week 3 of the acute infection stage to stage I | ***24*** *(2 weeks)* | [5] | *The duration is based on the assumption that during the remaining period of the acute infection the viral load (VL) is the same as during Stage I* | Fixed |  |
|  | $\theta_{1}$ | from stage I: asymptomatic to stage II: mild symptoms | ***1/1.19*** | [7] |  | Fixed |  |
|  | $\theta_{2}$ | from stage II: mild symptoms to stage III: more severe symptomatic HIV | ***1/3.0*** | [7] |  | Fixed |  |
|  | $\theta_{3}$ | from stage III: more severe symptoms to stage IV: AIDS | ***1/3.7*** | [7] |  | Fixed |  |
|  | *ε* | rate at which the HIV progression (in stages I-IV) decreases due to ART | ***0.1*** | [8] |  | Fixed |  |

* **“eclipse phase”** is the period after the transmission during which the infection dissemination in the systemic circulation has not yet occurred at detectable levels. ***Link****:* [*Detection of Acute HIV Infection | The Journal of Infectious Diseases | Oxford Academic (oup.com)*](https://academic.oup.com/jid/article/202/Supplement_2/S270/852813)

***Table B.*** HIV diagnostics

| **Parameter** | | **Description** | **Value** | **Source** | **Comments** | **Fixed/ estimate** | |
| --- | --- | --- | --- | --- | --- | --- | --- |
|  |  | **Active (screening) and passive (symptomatic) testing** | | | |  |  |
|  | *cov_ta_y_* | coverage: average screening (active) tests per year/all PWID pop | ***time varying*** |  | *assuming time-varying annual coverage levels for HIV testing based on data from the Republican Centre for Bloodborne Viral Hepatitis and HIV Control.* | Fixed |  |
|  | *cov_tp_y_* | coverage: average symptomatic (passive) tests per year/HIV+ PWID who developed symptoms | ***time varying*** |  | *assuming time-varying annual coverage levels for HIV testing based on data from the Republican Centre for Bloodborne Viral Hepatitis and HIV Control.* | Fixed |  |
|  | $\rho$ | correcting factor of overlapping in screening between Global Fund (GF) and USAID projects | ***time varying*** |  | *Refer to Table E* | Fixed |  |
|  | *test_2_* | proportion of positive cases at screening who take 2^nd^ test *(w/o interventions)* | ***0.7 (0.6-0.8)*** |  | *based on local expert opinions* | Fixed |  |
|  | *test_cnf_* | proportion of 2^nd^ test positives who take confirmatory tests (*w/o interventions)* | ***0.7 (0.6-0.8)*** |  | *based on local expert opinions* | Fixed |  |
|  |  | **Sensitivity of HIV tests** | | | | |  |
|  | ${Se}_{1}$ | sensitivity of rapid test (RT) | ***0.99*** | [9] | *Kyrgyz national guidelines for HIV diagnostics* | Fixed |  |
|  | ${Se}_{2}$ | sensitivity of enzyme-linked immunosorbent assay (ELISA) test | ***0.99*** | [9] | *Kyrgyz national guidelines for HIV diagnostics* | Fixed |  |
|  | $\delta$ | decrease in detecting positive cases (for each of the screening and confirmatory tests) during acute infection | ***0.5*** | [10], [6] | *Based on local expert opinions and evidence on the “window periods” for RT and Elisa tests*  *(1=no decrease, 0=full decrease)* | Fixed |  |
|  | *ta* | active testing rate | ***-*** |  | *Refer to Table H for the calculation.* |  |  |
|  | *tp* | passive testing rate | ***-*** |  | *Refer to Table H for the calculation.* |  |  |

***Table C.*** Antiretroviral treatment of HIV (ART)

| **Parameter** | | **Description** | **Value** | **Source** | **Comments** | **Fixed/ estimate** | |
| --- | --- | --- | --- | --- | --- | --- | --- |
|  |  | **PWID who immediately started ART after being diagnosed with HIV** | | | | |  |
|  | $t_{a1}$ | time for immediate launch of ART for stage I | ***2016*** | [11] | *Based on WHO guidelines and national HIV program* | Fixed |  |
|  | $\phi_{1}$ | Initial coverage with immediate ART for stage I | ***0.1*** |  | *Based on expert opinions* |  |  |
|  | $t_{a2}$ | time for immediate launch of ART for stage II | ***2013*** | [12] | *Based on WHO guidelines and national HIV program* | Fixed |  |
|  | $\phi_{2}$ | Initial coverage with immediate ART for stage I | ***0.2*** |  | *Based on expert opinions* |  |  |
|  | $t_{a3}$ | time for immediate launch of ART for stage III | ***2010*** | [13] | *Based on WHO guidelines and national HIV program* | Fixed |  |
|  | $\phi_{3}$ | initial coverage with immediate ART for stage III | ***0.5*** |  | *Based on expert opinions* |  |  |
|  | $t_{a4}$ | time for immediate launch of ART for stage IV | ***2005*** | [14], [15] | *Based on WHO guidelines and national HIV program* | Fixed |  |
|  | $\phi_{4}$ | initial coverage with immediate ART for stage III | ***0.6*** |  | *Based on expert opinions* |  |  |
|  |  | **Average time to start ART after being diagnosed at various stages if not linked to treatment immediately** | | | | |  |
|  | $\alpha_{1}$ | 1/average time to start ART after HIV is diagnosed at stage I | ***1/5*** |  | *Proxy (based on expert opinions and HIV progression periods)* | Fixed |  |
|  | $\alpha_{2}$ | 1/average time to start ART after HIV is diagnosed at stage II | ***1/3*** |  | *Proxy (based on expert opinions and HIV progression periods)* | Fixed |  |
|  | $\alpha_{3}$ | 1/average time to start ART after HIV is diagnosed at stage III | ***1/2*** |  | *Proxy (based on expert opinions and HIV progression periods)* | Fixed |  |
|  | $\alpha_{4}$ | 1/average time to start ART after HIV is diagnosed at stage IV | ***4*** *(3 months)* |  | *Proxy (based on expert opinions and HIV progression periods)* | Fixed |  |
|  |  | **Adherence to ART** | | | | |  |
|  | $\omega$ | rate of moving from ART state to non-ART state (1/average ART adherence period) | ***-*** |  | *No info/data* | Estimated |  |

***Table D.*** Prevention and ART adherence interventions

| **Parameter** | | **Description**  **Opioid Substitution Therapy (OST)** | **Value** | **Source** | **Comments** | **Fixed/ estimate** | |
| --- | --- | --- | --- | --- | --- | --- | --- |
|  | c__o_ | coverage with OST | ***0.04*** | [16] |  | Fixed |  |
|  | t__o_ | year of the launch of OST | ***2002*** |  |  | Fixed |  |
|  | e__ob_ | efficacy of OST in reducing the transmission ($\beta$) | ***0.54 (0.32-0.67)*** | [17] |  | Fixed |  |
|  | e__ow_ | efficacy of OST in improving adherence to ART (i.e. increasing $\gamma$) | ***0.69*** | [18] |  | Fixed |  |
|  | e__oa_ | efficacy of OST in reducing the time for starting ART (i.e. reducing $\alpha$and improving $\phi$) | ***0.50*** |  | *based on expert opinions* | Fixed |  |
|  |  | **Needle/syringe Exchange Program (NSP)** | |  |  |  |  |
|  | c__n_ | coverage with NSP | ***Time varying*** |  | *Refer to Table F* | Fixed |  |
|  | e__nb_ | efficacy of NSP in reducing the transmission ($\beta$) | ***0.66 (0.43-1.01)*** | [19] |  | Fixed |  |
|  |  | **Pre-exposure prophylaxis (PrEP)** | |  |  |  |  |
|  | c__p_ | coverage with PrEP | ***0.01*** | [20] |  | Fixed |  |
|  | t__p_ | year of the launch of PrEP | ***2019*** |  |  | Fixed |  |
|  | e__p_ | efficacy of PrEP in reducing the transmission (β) | ***0.48 (9.6-72.2)*** | [4] |  | Fixed |  |
|  |  | **Behaviour change interventions (Beh): peer support/education/etc.** | | | |  |  |
|  | *c__b_* | coverage with Beh | ***Time varying*** |  | *Refer to Table G* | Fixed |  |
|  | e__bw_ | efficacy of Beh in improving adherence to ART (i.e. increasing γ) | ***0.40*** | [21] | *based on evidence and experts' opinions* | Fixed |  |
|  | e__ba_ | efficacy of Beh in reducing the time for starting ART if not immediate linkage (i.e. reducing α and improving ϕ) | ***0.5*** |  | *Based on experts’ opinions* | Fixed |  |
|  | e__bb_ | efficacy of Beh in reducing HIV transmission (i.e. reducing $\beta$) | ***0.47 (0.24-0.62)*** | [22] |  | Fixed |  |

***Table E.*** Factor $\boldsymbol{(\rho)}$ to correct the overlapping in screening between GF and USAID projects

|  | | **Year** | $\boldsymbol{\rho}$ **factor values** | **Source** | **Comments** | |
| --- | --- | --- | --- | --- | --- | --- |
|  | 2016 | | 0.5 |  | *Assumption is based on expert opinions* |  |
|  | 2017 | | 0.5 |  | *Assumption is based on expert opinions* |  |
|  | 2018 | | 0.5 |  | *Assumption is based on expert opinions* |  |

***Table F.*** Needle/Syringe Exchange Programs

|  | | **Year** | **Number of tested persons** | **Source** | **Comments** | |
| --- | --- | --- | --- | --- | --- | --- |
|  | 2003 | | 0.12 | [23] |  |  |
|  | 2009 | | 0.67 | [24] |  |  |
|  | 2019 | | 0.60 |  | *Assumption is based on expert opinions* |  |
|  | 2020 | | 0.20 |  | *Assumption is based on expert opinions* |  |

***Table G.*** Behavioural interventions

|  | | **Year** | **Number of tested persons** | **Source** | **Comments** | |
| --- | --- | --- | --- | --- | --- | --- |
|  | 2002 | | 0.10 |  | *Assumption is based on expert opinions* |  |
|  | 2007 | | 0.40 |  | *Assumption is based on expert opinions* |  |
|  | 2009 | | 0.60 |  | *Assumption is based on expert opinions* |  |
|  | 2016 | | 0.70 |  | *Assumption is based on expert opinions* |  |
|  | 2020 | | 0.20 |  | *Assumption is based on expert opinions* |  |
|  | 2024 | | 0.40 |  | *Assumption is based on expert opinions* |  |

***Table H.*** Variables

| **Parameter** | | | **Description** | | **Formula** | **Comments** | | | |
| --- | --- | --- | --- | --- | --- | --- | --- | --- | --- |
|  | | *ta* | active testing rate | | *Se_1_ cov_ta_y_*Se_2_test_2_*Se_2_ test_cnf,_* | *Rt + RT/Elisa + Elisa* | | |  |
|  | | *tp* | passive testing rate | | *Se_2_ cov_tp_y_*Se_2_test_2_ *Se_2_ test_cnf,_* | *Rt/Elisa + RT/Elisa + Elisa* | | |  |
|  | | $\phi_{i\_n}$ | proportion of PWID who immediately started ART at stage_i_ after accounting for Beh and OST | | $\phi_{i\_Beh}$ *= e__ba_*c__b_**$\phi_{i}$  $\phi_{i\_OST}$ *= c__o_*e__oa_**$\phi_{i}$  $\phi_{i\_n}$ *=* $\phi_{i}+$ *(*$\phi_{i\_Beh}$ *,* $\phi_{i\_OST}$*)* | *after accounting for Beh and OST* | | |  |
|  | | $\alpha_{i\_n}$ | 1/average time to start ART after HIV is diagnosed at stage_i_ (if not linked immediately) after accounting for Beh and OST | | $\alpha_{i\_Beh}$ *= e__ba_*c__b_**$\alpha_{i}$  $\alpha_{i\_OST}$ *= e__oa_*c__o_*$*\alpha_{i}$  $\alpha_{i\_n}$ *=* $\alpha_{i}$ *+ (*$\alpha_{i\_Beh,} \alpha_{i\_OST}$*)* | *after accounting for Beh and OST* | | |  |
|  | | $\omega_{n}$ | adherence to ART: moving rate from ART status to non-ART status after accounting for Beh and OST | | $\omega_{n}=\omega$ *(1- c__o_*e__ow_(t>=t__o_))*(1-c__b_*e__bw_)* | *after accounting for Beh and OST* | | |  |
|  |  | | | **Force of infection:** $\boldsymbol{\lambda=}\boldsymbol{\beta}_{\boldsymbol{n}}\boldsymbol{*}\frac{\boldsymbol{I}}{\boldsymbol{N}}$ | | |  |  | |
|  | | $\sum Ac$ | non-treated I: acute infection periods | | $\iota_{a_{1}}$*(Au_1_)+ Au_2_ + Ad_2_ +* $\iota_{a_{3}}$*(Au_3_+Ad_3_)* | | | |  |
|  | | $\sum NTr$ | non-treated I: with reduced infectiousness if not acute infection | | $\iota_{1\_2}$*(U_1_+U_2_+D_1_+D_2_) +* $\iota_{a_{3}}$*(U_3_+D_3_) +* $\iota_{a_{4}}$*(U_4_+D_4_)* | | | |  |
|  | | $\sum Tr$ | treated I: with reduced infectiousness if on ART | | $\gamma$*(Atr_2_ + Atr_3_ +* $\iota_{1\_2}$*(Tr_1_+Tr_2_) +* $\iota_{a_{3}}$*Tr_3_ +* $\iota_{a_{4}}$*Tr_4_)* | | | |  |
|  | | $I$ | total population | | $\sum Ac+$ $\sum NTr+$ $\sum Tr$ |  | | |  |
|  | | $\beta_{n}$ | $\beta_{n}$ after accounting for PrEP, OST, NSP and Beh | | $\beta$*(1-c__p_*e__p_)*(1-c__o_*e__ob_)*(1-c__n_*e__nb_) )*(1-c__b_*e__bb_)* | | | |  |
|  | |  |  | |  |  | | |  |

**References:**

1. UNDP G. Bio-behavioral survey among people who inject drugs in the Kyrgyz Republic. 2016.

2. Morgan D, Mahe C, Mayanja B, Okongo JM, Lubega R, Whitworth JAG. HIV-1 infection in rural Africa: is there a difference in median time to AIDS and survival compared with that in industrialized countries? AIDS. 2002;16(4).

3. Zwahlen; M, Egger M. Progression and mortality of untreated HIV-positive individuals living in resource-limited settings: Update of literature review and evidence synthesis: UNAIDS; 2006 [Available from: <https://data.unaids.org/pub/periodical/2006/zwahlen_unaids_hq_05_422204_2007_en.pdf>.

4. Choopanya K, Martin M, Suntharasamai P, Sangkum U, Mock PA, Leethochawalit M, et al. Antiretroviral prophylaxis for HIV infection in injecting drug users in Bangkok, Thailand (the Bangkok Tenofovir Study): a randomised, double-blind, placebo-controlled phase 3 trial. The Lancet. 2013;381(9883):2083-90.

5. Selik RM, Linley L. Viral Loads Within 6 Weeks After Diagnosis of HIV Infection in Early and Later Stages: Observational Study Using National Surveillance Data. JMIR Public Health Surveill. 2018;4(4):e10770.

6. Deeks SG, Overbaugh J, Phillips A, Buchbinder S. HIV infection. Nature Reviews Disease Primers. 2015;1(1):15035.

7. Lodi S, Phillips A, Touloumi G, Geskus R, Meyer L, Thiébaut R, et al. Time From Human Immunodeficiency Virus Seroconversion to Reaching CD4+ Cell Count Thresholds <200, <350, and <500 Cells/mm3: Assessment of Need Following Changes in Treatment Guidelines. Clinical Infectious Diseases. 2011;53(8):817-25.

8. Sucharitakul K, Boily MC, Dimitrov D, Mitchell KM. Influence of model assumptions about HIV disease progression after initiating or stopping treatment on estimates of infections and deaths averted by scaling up antiretroviral therapy. PLoS One. 2018;13(3):e0194220.

9. MoH. Guidlines for laboratory diagnostics of HIV infection. Ministry of Health of the Kyrgyz Republic, "AIDS" Republican Centre. 2017.

10. Chu; C, A.Selwyn P. Diagnosis and Initial Management of Acute HIV Infection. Am Fam Physician. 2010;81(10):1239-44.

11. WHO. Consolidated guidelines on the use of antiretroviral drugs for treating and preventing HIV infection: recommendations for a public health approach – 2nd ed. 2016.

12. World Health Organisation. Consolidated guidelines on the use of antiretroviral drugs for treating and preventing HIV infection: recommendations for a public health approach. 2013.

13. WHO. Antiretroviral therapy for HIV infection in adults and adolescents: recommendations for a public health approach. 2010 revision. 2010.

14. WHO. Scaling up antiretroviral therapy in resource-limited settings. Guidelines for a public health approach 2002.

15. WHO. Country profile. Kyrgyzstan. 2005.

16. UNAIDS. Kyrgyzstan country profile. 2022 [Available from: <https://www.unaids.org/en/regionscountries/countries/kyrgyzstan>.

17. MacArthur GJ, Minozzi S, Martin N, Vickerman P, Deren S, Bruneau J, et al. Opiate substitution treatment and HIV transmission in people who inject drugs: systematic review and meta-analysis. BMJ : British Medical Journal. 2012;345:e5945.

18. Low AJ, Mburu G, Welton NJ, May MT, Davies CF, French C, et al. Impact of Opioid Substitution Therapy on Antiretroviral Therapy Outcomes: A Systematic Review and Meta-Analysis. Clin Infect Dis. 2016;63(8):1094-104.

19. Aspinall EJ, Goldberg DJ, Weir A, Van Velzen E, Palmateer N, Hutchinson SJ, et al. Are needle and syringe programmes associated with a reduction in hiv transmission among people who inject drugs: A systematic review and meta-analysis. International Journal of Epidemiology. 2014;43(1):235-48.

20. MoH. A bio-behavioural study on HIV infection among people who inject drugs and men who have sex with men in the Kyrgyz Republic. 2022.

21. Miller WC, Hoffman IF, Hanscom BS, Ha TV, Dumchev K, Djoerban Z, et al. A scalable, integrated intervention to engage people who inject drugs in HIV care and medication-assisted treatment (HPTN 074): a randomised, controlled phase 3 feasibility and efficacy study. Lancet. 2018;392(10149):747-59.

22. Booth RE, Davis JM, Dvoryak S, Brewster JT, Lisovska O, Strathdee SA, et al. HIV incidence among people who inject drugs (PWIDs) in Ukraine: results from a clustered randomised trial. The Lancet HIV. 2016;3(10):e482-e9.

23. Wolf D. Harm reduction in Kyrgyzstan: Saving the lives. Снижение Вреда в Кыргызстане: Спасая жизни. 2005.

24. State Programme on stabilization of HIV epidemic in the Kyrgyz Republic for 2012-2016. Government of the Kyrgyz Republic.; 2012.
