# Supplementary material for: Potential reasons for the decline of new HIV cases among people who inject drugs (PWID) in Kyrgyzstan
Source: PLoS One. 2026 May 26;21(5):e0348970. doi: 10.1371/journal.pone.0348970 (PMC13210176; doi:10.1371/journal.pone.0348970)
Supplement: S2 File — (DOCX) [file pone.0348970.s002.docx]

**S2.**

**Fig 1**. Model framework


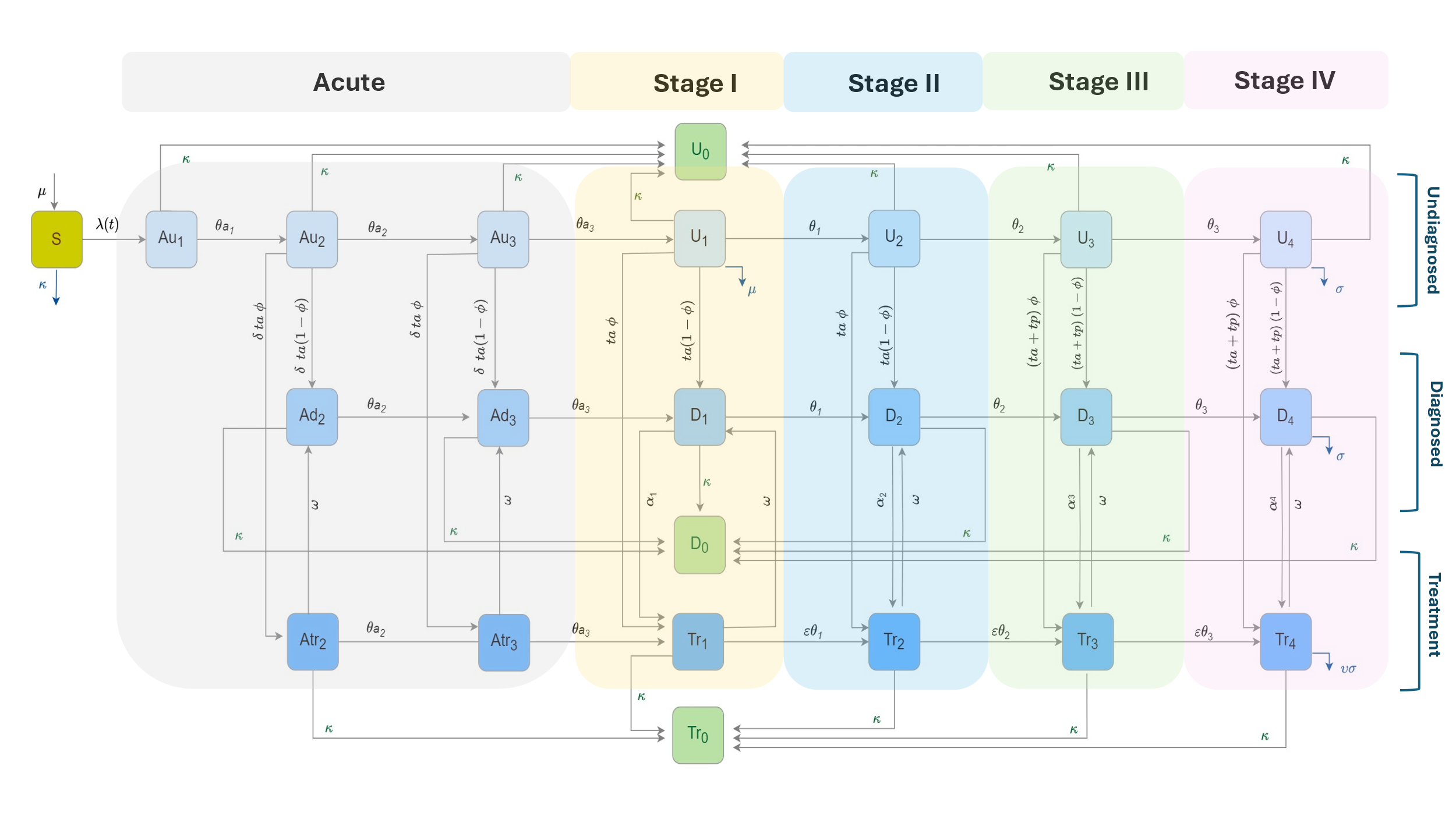


**Equations:**

$$\frac{dS}{dt}=\mu N-(t)S-\kappa S$$

$$\frac{d{Au}_{1}}{dt}=\left( t \right)S-\left( \kappa+ \theta a_{1} \right){Au}_{1}$$

$$\frac{d{Au}_{2}}{dt}=\theta a_{1}{Au}_{1}-\delta ta {Au}_{2}-\left( \kappa+ \theta a_{2} \right){Au}_{2}$$

$$\frac{d{Au}_{3}}{dt}=\theta a_{2}{Au}_{2}-\delta ta {Au}_{3}-\left( \kappa+ \theta a_{3} \right){Au}_{3}$$

$$\frac{dU_{1}}{dt}= \theta a_{3}{Au}_{3}-taU_{1}-\left( \kappa+ \theta_{1} \right)U_{1}$$

$$\frac{dU_{2}}{dt}=\theta_{1}U_{1}-taU_{2}-\left( \kappa+ \theta_{2} \right)U_{2}$$

$$\frac{dU_{3}}{dt}=\theta_{2}U_{2}- \left( ta+tp \right)U_{3}-\left( {\kappa+ \theta}_{3} \right)U_{3}$$

$$\frac{dU_{4}}{dt}=\theta_{3}U_{3}- \left( ta+tp \right)U_{4}-\left( \kappa+\sigma\right)U_{4}$$

$$\frac{dU_{0}}{dt}=\kappa{(Au}_{1}+{Au}_{2}+ {Au}_{3}+U_{1}+U_{2}+U_{3}+U_{4})$$

$$\frac{d{Ad}_{2}}{dt}=\delta ta \left( 1-\phi\right){Au}_{2}+ \omega{Atr}_{2}-\left( \kappa+\theta a_{2} \right){Ad}_{2}$$

$$\frac{d{Ad}_{3}}{dt}=\theta a_{2}{Ad}_{2}+\delta ta \left( 1-\phi\right){Au}_{2}+ \omega{Atr}_{3}-\left( \kappa+\theta a_{3} \right){Ad}_{3}$$

$$\frac{dD_{1}}{dt}=\theta a_{3}{Ad}_{3}+ ta \left( 1-\phi\right)U_{1}+ \omega{Tr}_{1}-\left( \alpha_{1}+ \kappa+ \theta_{1} \right)D_{1}$$

$$\frac{dD_{2}}{dt}= \theta_{1}D_{1}+ ta \left( 1-\phi\right)U_{2}+ \omega{Tr}_{2}-\left( \alpha_{2}+ \kappa+ \theta_{2} \right)D_{2}$$

$$\frac{dD_{3}}{dt}= \theta_{2}D_{2}+(ta+tp) \left( 1-\phi\right)U_{3}+ \omega{Tr}_{3}-\left( \alpha_{3}+\kappa+ \theta_{3} \right)D_{3}$$

$$\frac{dD_{4}}{dt}= \theta_{3}D_{3}+(ta+tp) \left( 1-\phi\right)U_{4}+ \omega{Tr}_{4}-\left( \alpha_{4}+ \kappa+ \sigma\right)D_{4}$$

$$\frac{dD_{0}}{dt}=\kappa({Ad}_{2}+ {Ad}_{3}+D_{1}+D_{2}+D_{3}+D_{4})$$

$$\frac{d{Atr}_{2}}{dt}=\delta ta \phi{Au}_{2}-\left( \omega+ \kappa+ \theta a_{2} \right){Atr}_{2}$$

$$\frac{d{Atr}_{3}}{dt}=\delta ta \phi{Au}_{3}-\left( \omega+ \kappa+ \theta a_{3} \right){Atr}_{3}$$

$$\frac{d{Tr}_{1}}{dt}=\theta a_{3}{Atr}_{3}+ ta \phi U_{1} + \alpha_{1}D_{1}-\left( \omega+ \kappa+ \varepsilon\theta_{1} \right){Tr}_{1}$$

$$\frac{d{Tr}_{2}}{dt}=\varepsilon\theta_{1}{Tr}_{1}+ta \phi U_{2} + \alpha_{2}D_{2}-\left( \omega+ \kappa+ \varepsilon\theta_{2} \right){Tr}_{2}$$

$$\frac{d{Tr}_{3}}{dt}=\varepsilon\theta_{2}{Tr}_{2}+\left( ta+tp \right)\phi U_{3} + \alpha_{3}D_{3}-\left( \omega+\kappa+ \varepsilon\theta_{3} \right){Tr}_{3}$$

$$\frac{d{Tr}_{4}}{dt}=\varepsilon\theta_{3}{Tr}_{3}+(ta+tp) \phi U_{4} + \alpha_{4}D_{4}-(\omega+\kappa+\upsilon\sigma){Tr}_{4}$$

$$\frac{d{Tr}_{0}}{dt}=\kappa({Atr}_{2}+ {Atr}_{3}+{Tr}_{1}+{Tr}_{2}+{Tr}_{3}+{Tr}_{4})$$

**Note:** $U_{0}, D_{0}, {Tr}_{0}$ denote compartments representing individuals who left the PWID population either before or after being registered in the national HIV registry

**Box 1.** Definitions of HIV progression stages^[[1]](#footnote-1)^

| **Acute stage:** | $\mathbf{Au}_{\mathbf{1}}\mathbf{,}\mathbf{Au}_{\mathbf{2}}\mathbf{,}\mathbf{Au}_{\mathbf{3}}\mathbf{,}$  $\mathbf{Ad}_{\mathbf{2}}\mathbf{,}\mathbf{Ad}_{\mathbf{3}}\mathbf{, Atr}$ | The acute infection period has been subdivided into several compartments, as these differ in viral load levels (Appendix 1, Table A) |
| --- | --- | --- |
| **Stage I:** | $\mathbf{U}_{\mathbf{1}}\mathbf{,}\mathbf{D}_{\mathbf{1}}\mathbf{,}\mathbf{Tr}_{\mathbf{1}}\boldsymbol{,}$ | No symptoms (asymptomatic) and not significant immunosuppression (CD4>500 copies/ml); |
| **Stage II:** | $\mathbf{D}_{\mathbf{2}}\mathbf{,}\mathbf{Tr}_{\mathbf{2}}$ | Mild symptoms (moderate weight loss, recurrent respiratory tract infections and dermatological infections) and/or mild immunosuppression (CD4 350-499 copies/ml); |
| **Stage III:** | $\mathbf{U}_{\mathbf{3}}\mathbf{,}\mathbf{D}_{\mathbf{3}}\mathbf{,}\mathbf{Tr}_{\mathbf{3}}$ | Advanced symptoms (severe weight loss, chronic diarrhoea, oral candidiasis, TB, severe bacterial infections, etc) and/or advanced immunosuppression (CD4 200-349 copies/ml); |
| **Stage IV:** | $\mathbf{U}_{\mathbf{4}}\mathbf{,}\mathbf{D}_{\mathbf{4}}\mathbf{,}\mathbf{Tr}_{\mathbf{4}}$ | AIDS-related condition (extrapulmonary TB, Kaposi sarcoma etc.) and/or severe immunosuppression (CD4<200 copies/ml) |

**Model calibration**

The model was calibrated using available data on reported annual HIV cases among PWID for the period of 2002-2023, employing an optimisation approach to minimise the negative log-likelihood (NLL). The likelihood function was based on the Poisson distribution, with the model's estimated HIV incidence representing the average annual rate of new infections. This model calibration approach allowed for the estimation of the transmission coefficient ($\boldsymbol{\beta}$) and the transition rate from the ART state to the non-ART state ($\boldsymbol{\omega}$), with corresponding 95% confidence intervals (CIs) obtained through the NLL minimisation process.

To enhance robustness, the model was fitted across a range of PWID population replenishment scenarios, where population entry rates ($\boldsymbol{\mu}$) were set equal to, lower than, or higher than the population exit rates ($\boldsymbol{\kappa}$), **Fig. 2a–b**.

The Akaike Information Criterion (AIC) was used for model selection and comparison, with AIC calculated as ***AIC=2n_p_ +2NLL***, where 𝑛_𝑝_ denotes the number of estimated parameters and NLL is the negative log-likelihood. Models with an AIC difference from the best-performing model (*ΔAIC_𝑖_ = AIC_𝑖_ – AIC_min_*) of 4 or more are considered to have substantially less support (Table A).

**Table A.** Model selection using AIC approach

| Model ID | $\mu$*=* | Number of estimated parameters (n_p_) | NLL | AIC_𝑖_ | ΔAIC_𝑖_ | Support  (***Yes*** for ΔAIC_𝑖_<4, otherwise ***No***) |
| --- | --- | --- | --- | --- | --- | --- |
| Model 1 | $\kappa$-0.035 | 2 | 169.99 | 343.98 | 3.0 | yes |
| Model 2 | $\kappa$-0.030 | 2 | 169.71 | 343.42 | 2.5 | yes |
| Model 3 | $\kappa$-0.025 | 2 | 169.53 | 343.06 | 2.1 | yes |
| Model 4 | $\kappa$-0.020 | 2 | 169.17 | 342.34 | 1.4 | yes |
| Model 5 | $\kappa$-0.015 | 2 | 168.79 | 341.58 | 0.6 | yes |
| Model 6 | $\kappa$-0.010 | 2 | 168.48 | 340.96 | 0.0 | yes |
| Model 7 | $\kappa$-0.005 | 2 | 169.04 | 342.08 | 1.1 | yes |
| Model 8 | $\kappa$-0.000 | 2 | 170.84 | 345.68 | 4.7 | no |
| Model 9 | $\kappa$+0.005 | 2 | 171.28 | 346.56 | 5.6 | no |

Based on the above, the population replenishment scenarios with a good-fitting range from $\mu$ = $\kappa$- 0.035 to $\mu$ = $\kappa$- 0.005.

Further validation of the model with the annual HIV-attributable death data reported by the National AIDS Centre revealed an underestimation of modelled deaths for the scenario where ($\kappa-$ 0.015) ≥ $\mu$ and an overestimation for $\mu$ ≤ ($\kappa$ - 0.035). Validation with the number of registered PWID, reported by the National Narcology Centre, showed an exact match for $\mu$≤($\kappa-$0.035), suggesting an underestimation of the population due to various social, cultural, and legal barriers that prevent some PWID from registering with drug treatment hospitals.

Considering the above, the population replenishment scenarios where ($\kappa$– 0.030) ≤ $\mu$ ≤ ($\kappa$ – 0.020) showed the best model fit. Within this range, the scenario with $\mu$ = $\kappa$ – 0.020 produced the lowest objective function value (OFV = 169.17). Although differences in OFV across these scenarios were minimal, this scenario was selected for the subsequent testing of hypotheses regarding the observed decline in reported HIV cases among PWID in Kyrgyzstan.

**Fig 2a. Model calibration and population dynamics in model scenarios with baseline interventions and *μ <*** $\boldsymbol{\kappa}$***,*** $\boldsymbol{\mu}$ ***≈*** $\boldsymbol{\kappa}$***,*** $\boldsymbol{\mu}$ ***>*** $\boldsymbol{\kappa}$

|  | Estimated average and 95% CI | | | | | | | | |
| --- | --- | --- | --- | --- | --- | --- | --- | --- | --- |
| Transmission coefficient ($\boldsymbol{\beta}$)  Transition from ART to non-ART state ($\boldsymbol{\omega}$**)** | 2.27  (2.26-2.28)  16.28  (9.90-20.67) | 2.27  (2.26-2.29)  9.90  (6.03-12.25) | 2.29  (2.26-2.32)  1.86  (1.48-3.03) | 2.29  (2.28-2.31)  0.66  (0.40-1.31) | 2.30  (2.29-2.31)  0.22  (0.12-0.40) | 2.30  (2.29-2.31)  0.003  (0.001 – 0.007) | 2.29  (2.28-2.30)  0.000  (3e-4–6e-6) | 2.29  (2.28-2.29)  0.000  (1e-5 – 3e-6) | 2.28  (2.27-2.29)  0.000  (4e-10 – 6e-16) |
| Objective function value (OFV) | 169.99 | 169.71 | 169.53 | 169.17 | 168.79 | 168.48 | 169.04 | 170.84 | 171.28 |
| Entry rate **(**$\boldsymbol{\mu}$**) =** | $\boldsymbol{\kappa}$**-0.035** | $\boldsymbol{\kappa}$***-*0.030** | $\boldsymbol{\kappa}$**-0.025** | $\boldsymbol{\kappa}$**-0.020** | $\boldsymbol{\kappa}$***-*0.015** | $\boldsymbol{\kappa}$**-0.010** | $\boldsymbol{\kappa}$**-0.005** | $\boldsymbol{\kappa}$ | $\boldsymbol{\kappa}$**+0.005** |
|  |  |  |  |  |  |  |  |  |  |
|  | $\boldsymbol{\mu<\kappa}$ $\boldsymbol{\mu\approx\kappa}$ $\boldsymbol{\mu>\kappa}$ | | | | | | | | |
| MLE OPTIM: Model vs. actual reported  HIV cases/year | **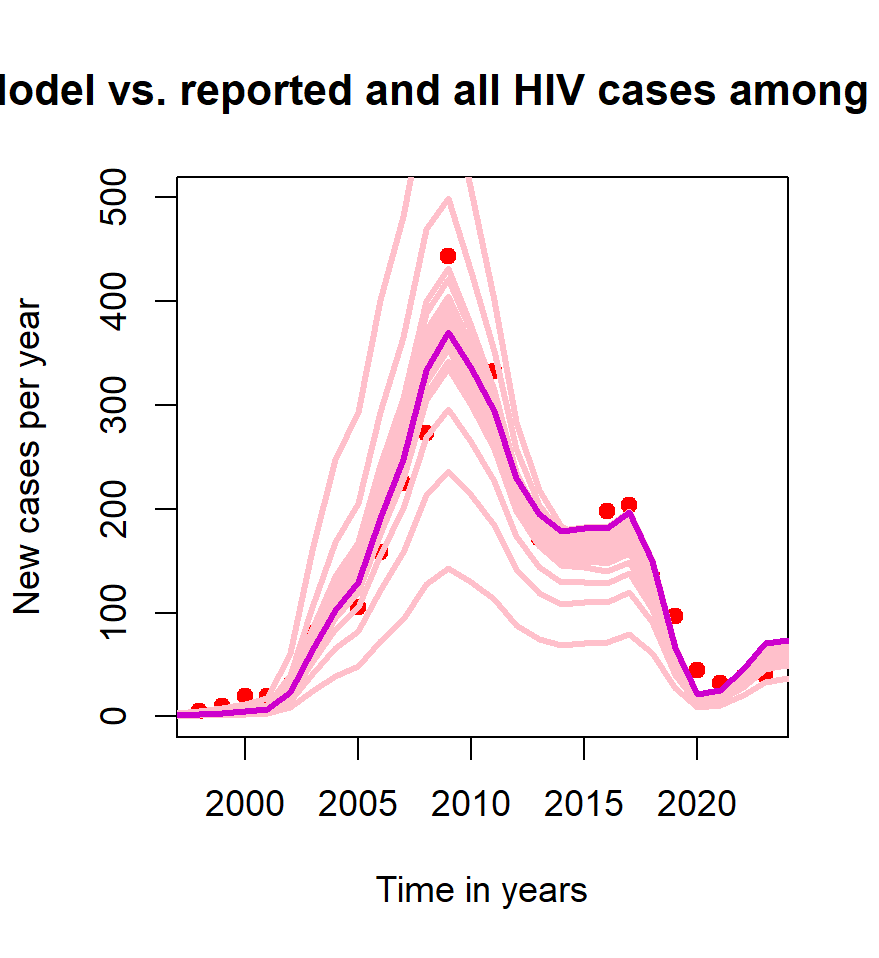** | **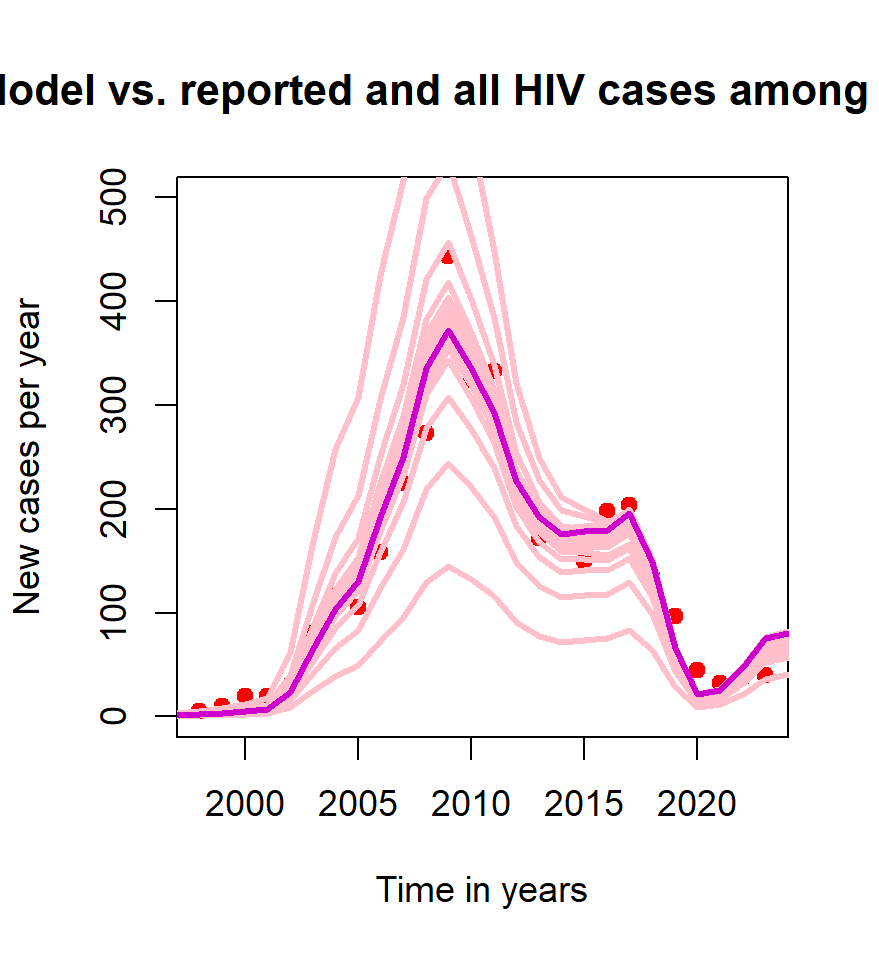** | **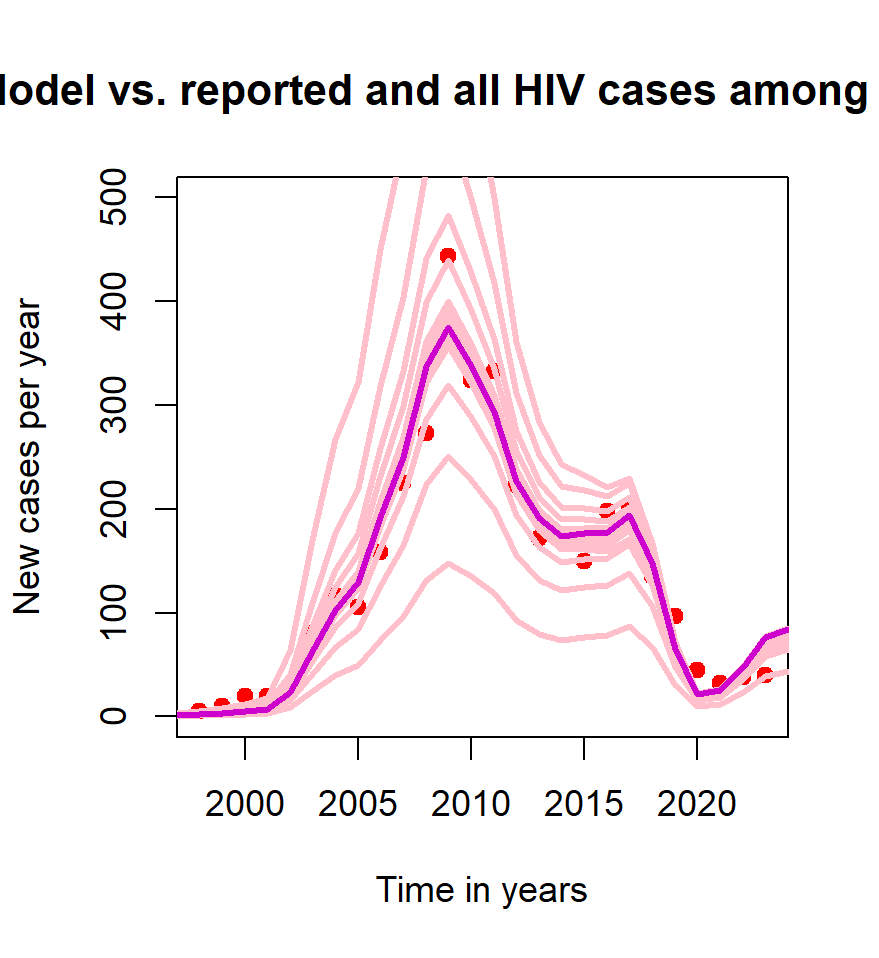** | **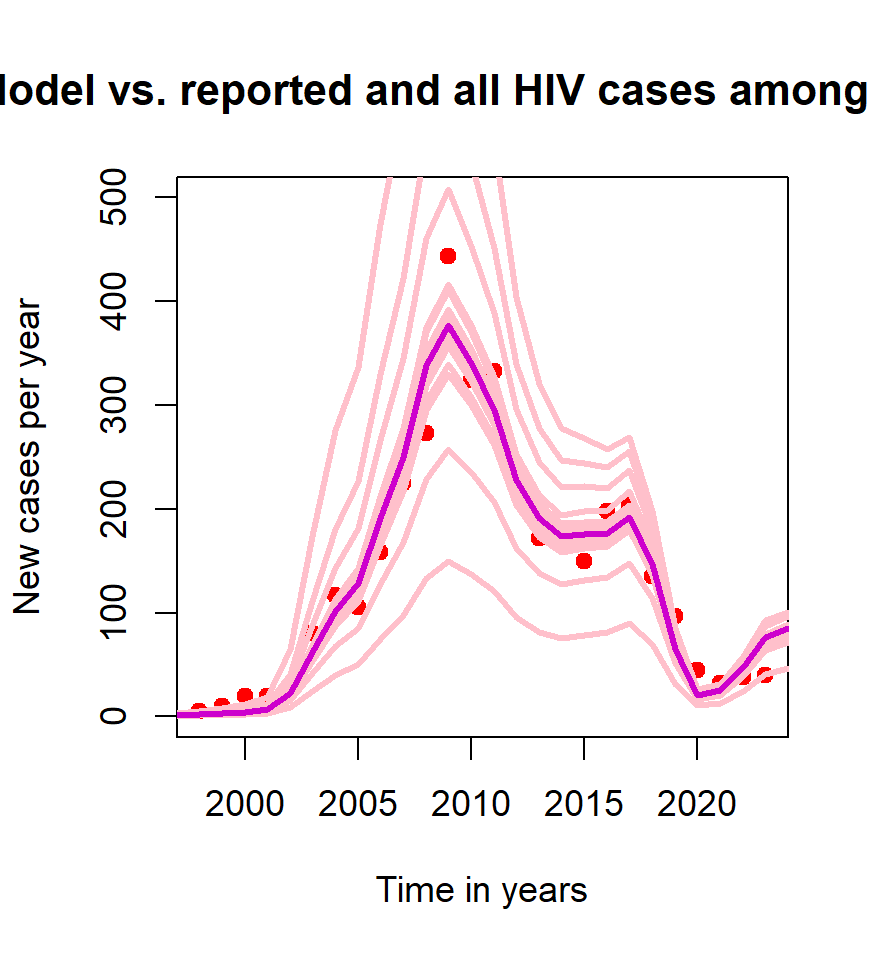** | **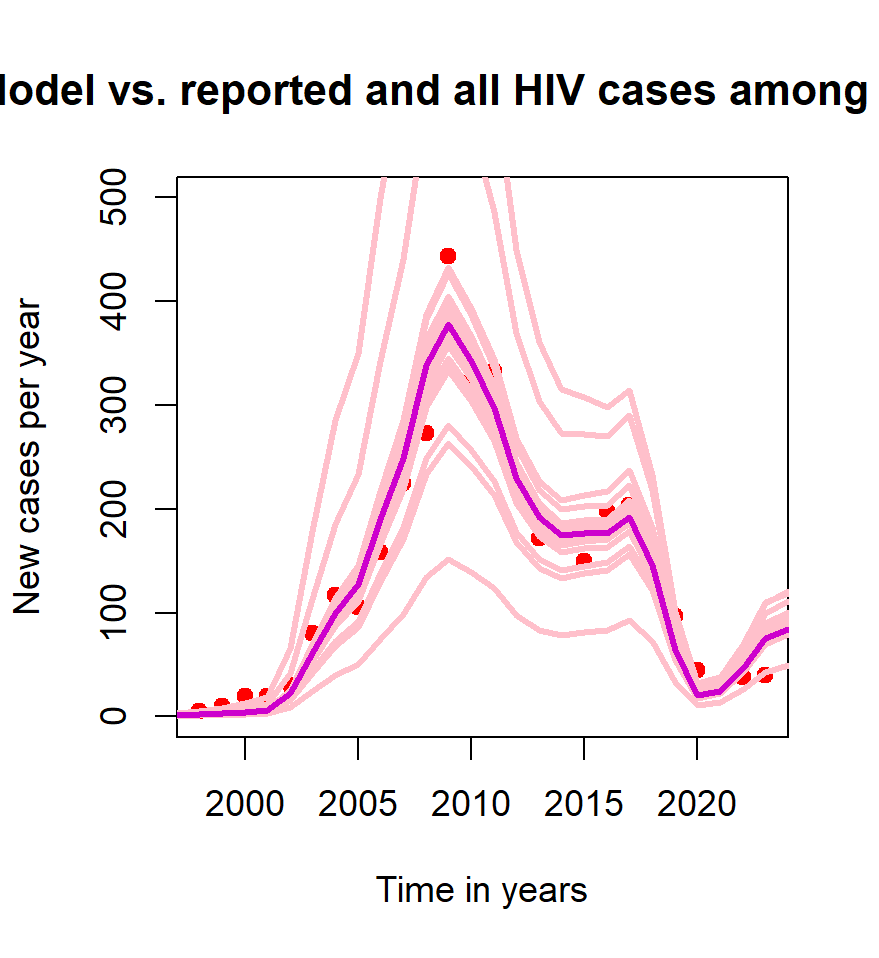** | **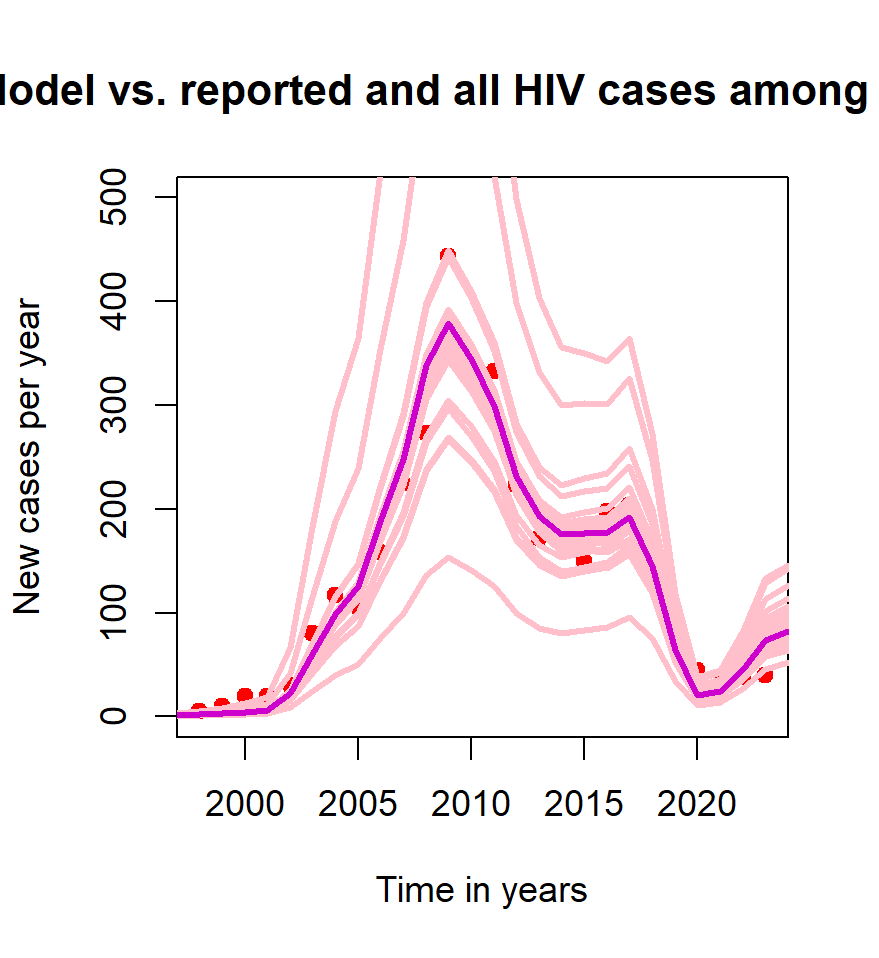** | **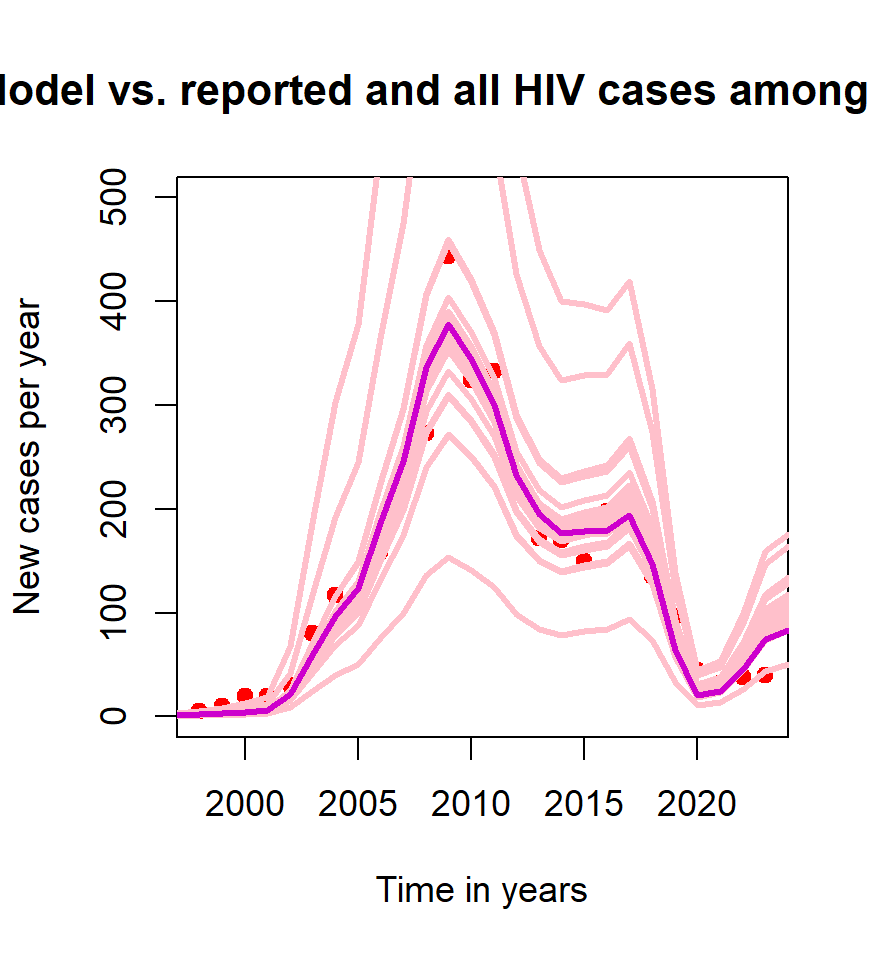** | **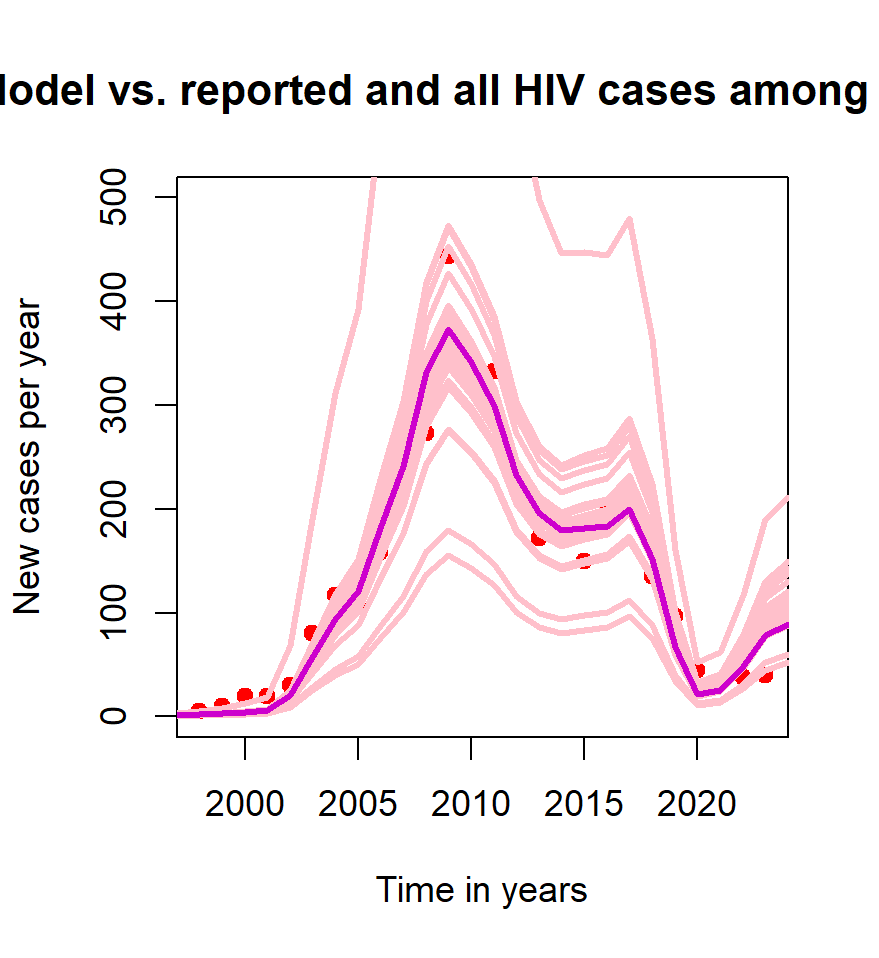** | **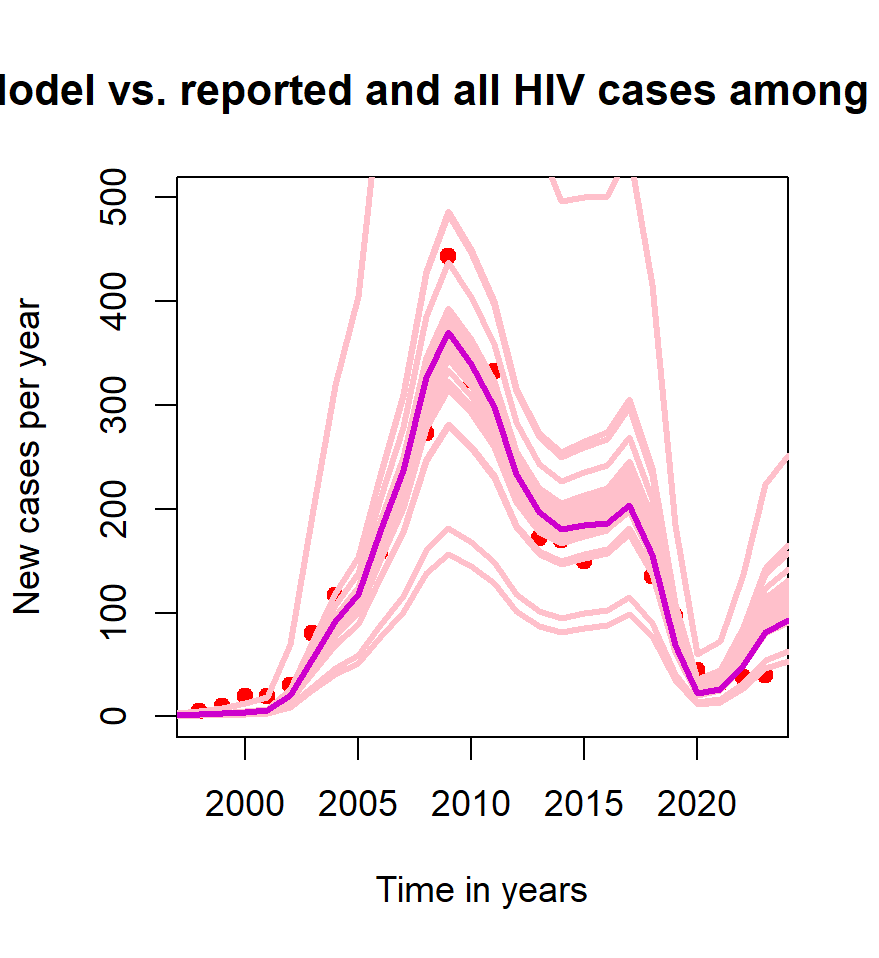** |

| **Fig 2b. Model validation with additional data** | | | | | | | | | | |
| --- | --- | --- | --- | --- | --- | --- | --- | --- | --- | --- |
| Entry rate **(**$\boldsymbol{\mu}$**) =** |  | $\boldsymbol{\kappa}$**-0.035** | $\boldsymbol{\kappa}$***-*0.030** | $\boldsymbol{\kappa}$**-0.025** | $\boldsymbol{\kappa}$**-0.020** | $\boldsymbol{\kappa}$***-*0.015** | $\boldsymbol{\kappa}$**-0.010** | $\boldsymbol{\kappa}$**-0.005** | $\boldsymbol{\kappa}$ | $\boldsymbol{\kappa}$**+0.005** |
| Model vs. actually reported HIV attributable mortality/year  Y-axis: Model vs. actually reported HIV attributable mortality/year |  | **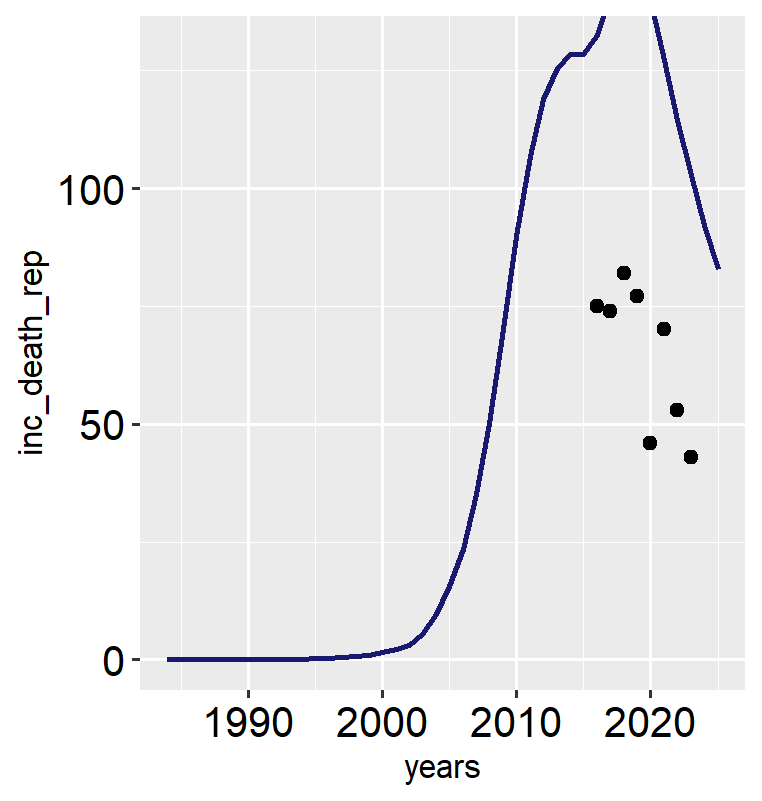** | **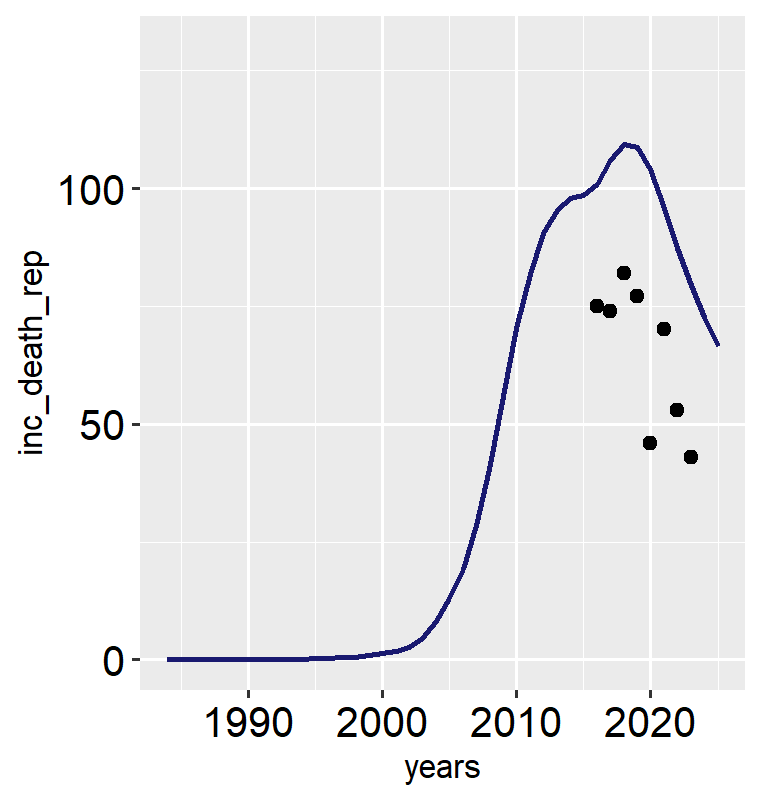** | **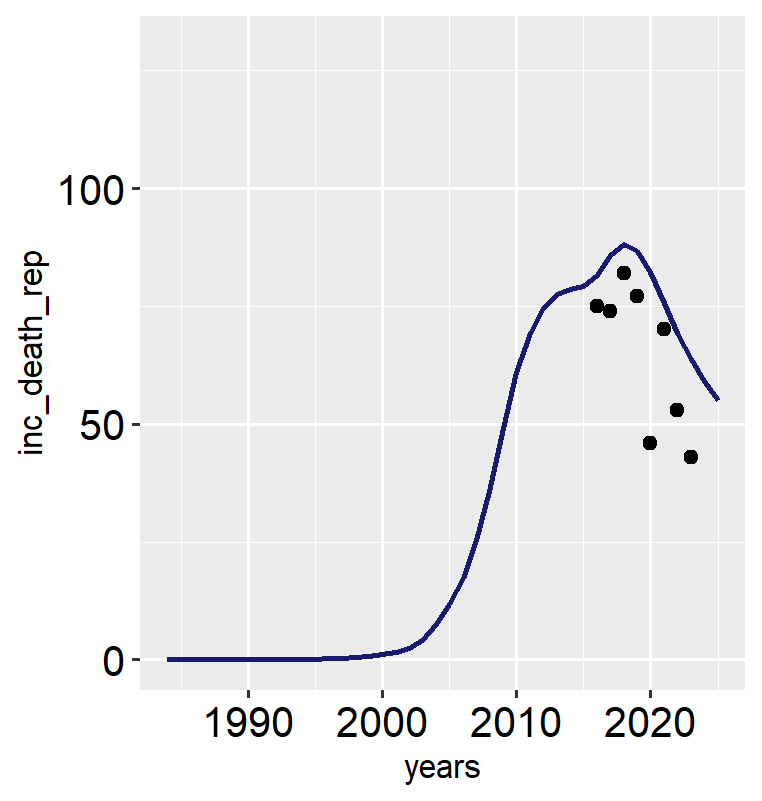** | **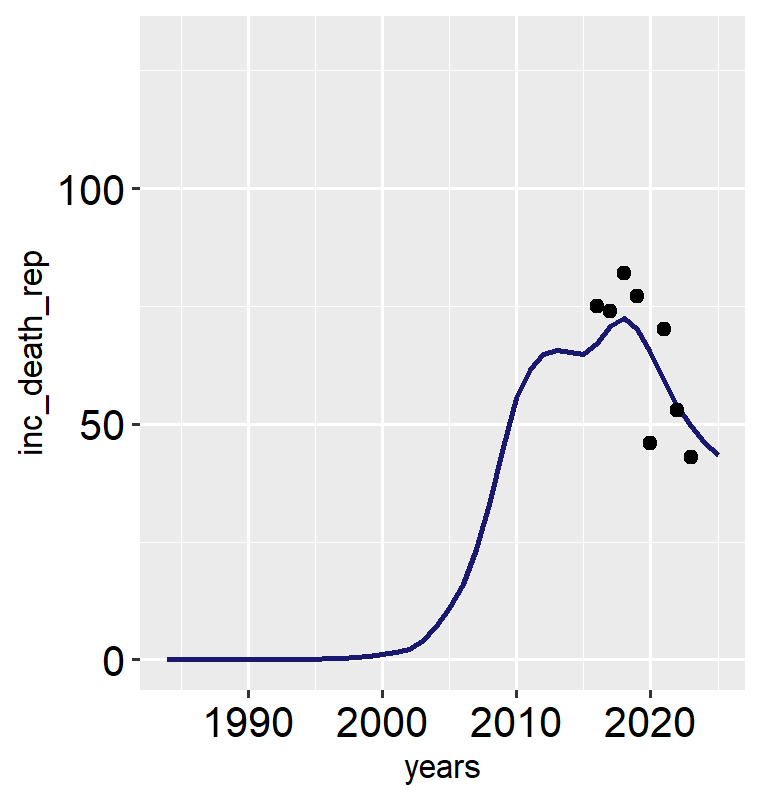** | **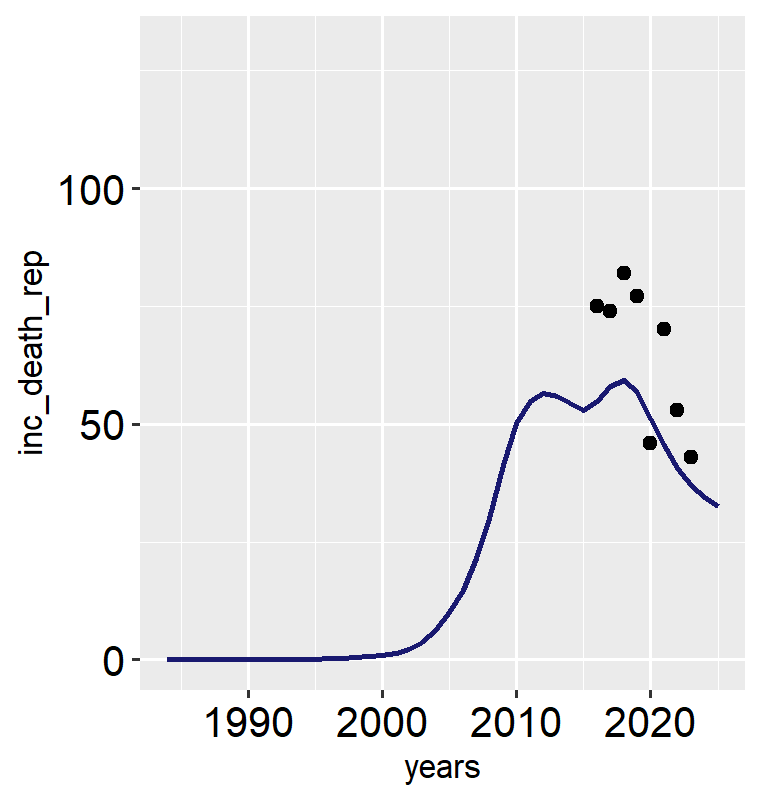** | **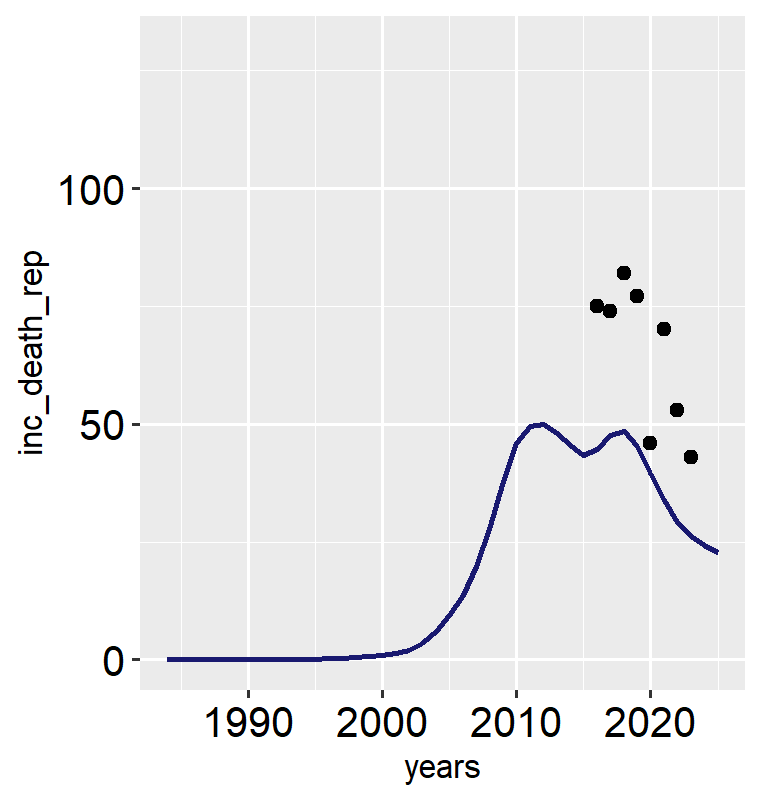** | **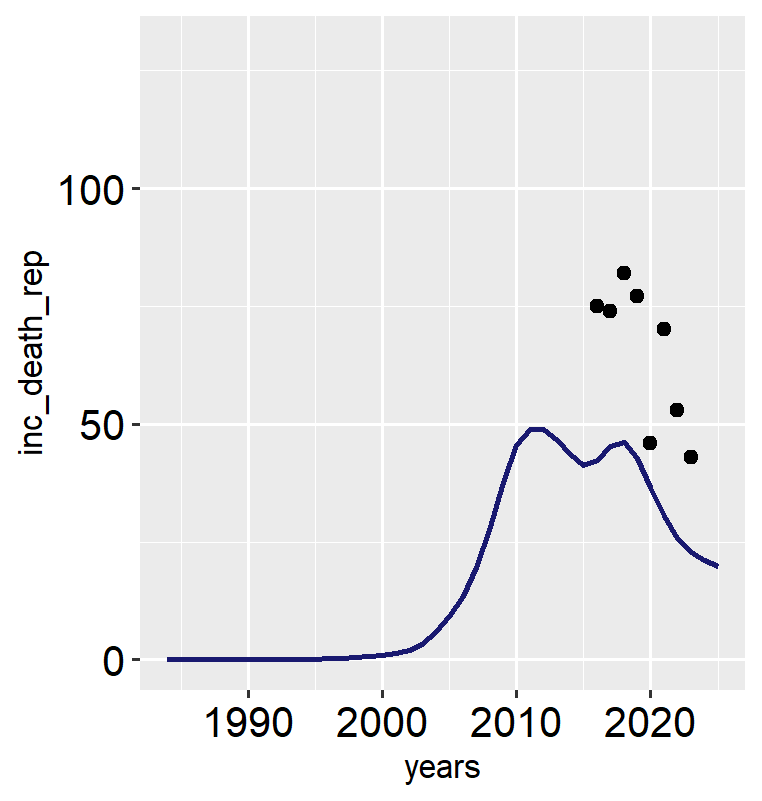** | **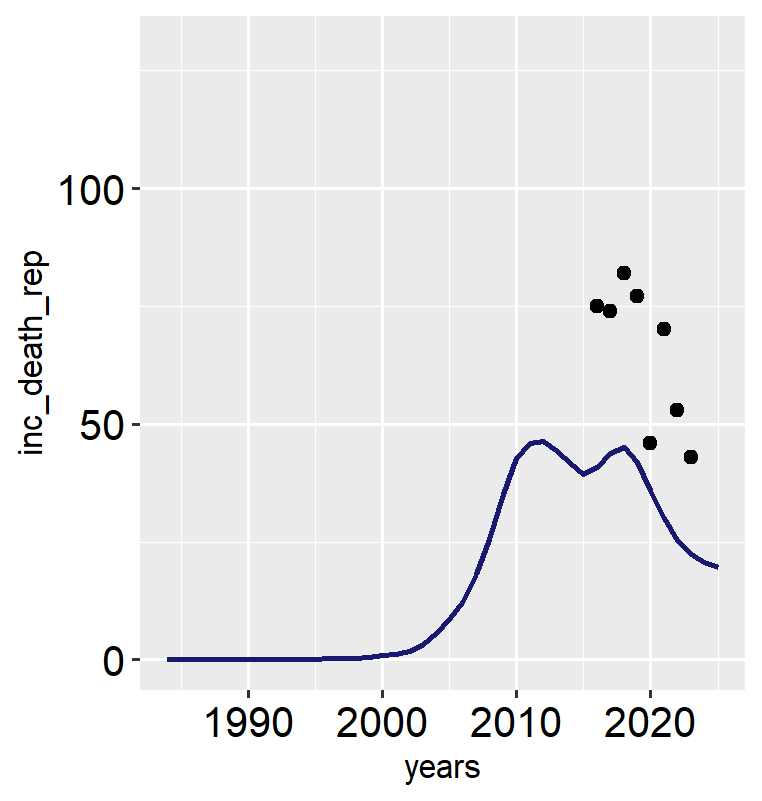** | **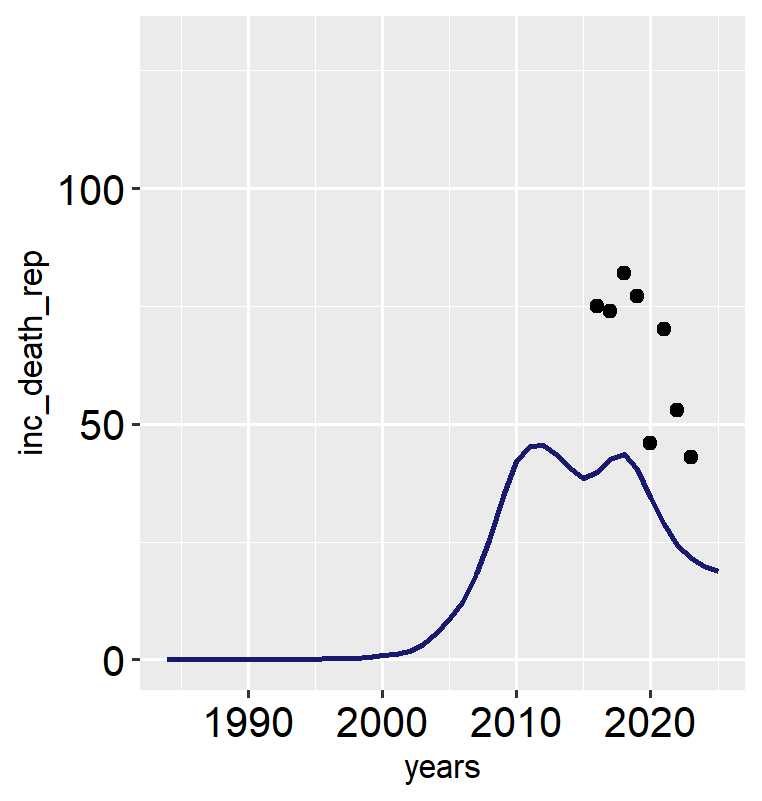** |
| Model vs. reported PWID population (registered with Narcology) | Y-axis: PWID population | **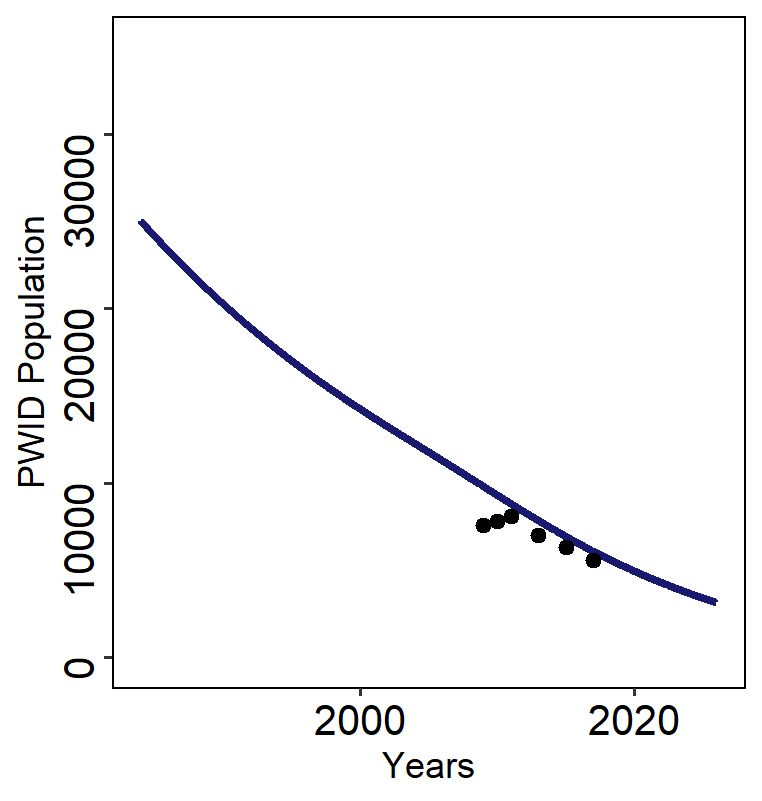** | **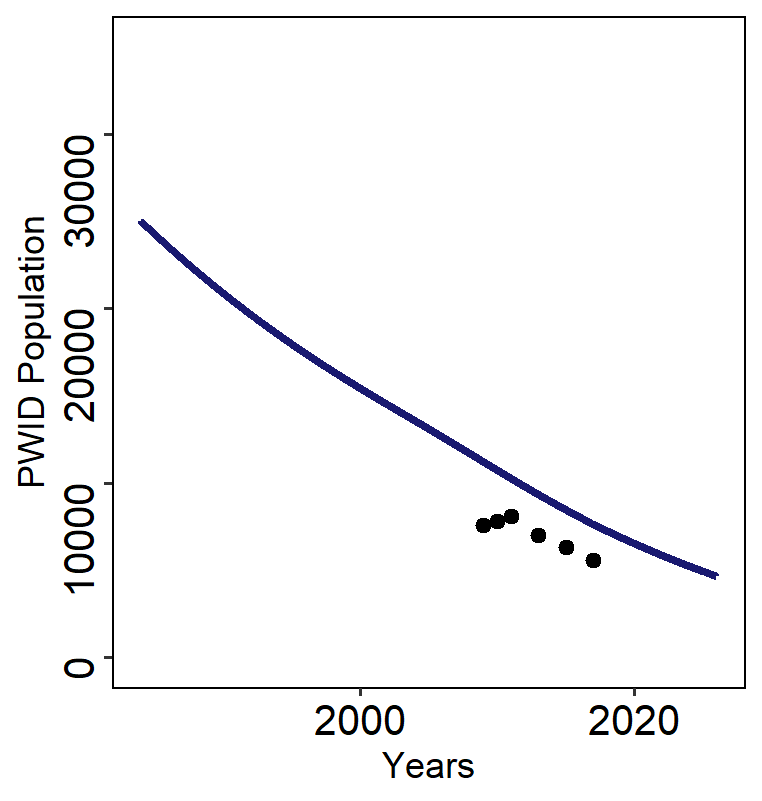** | **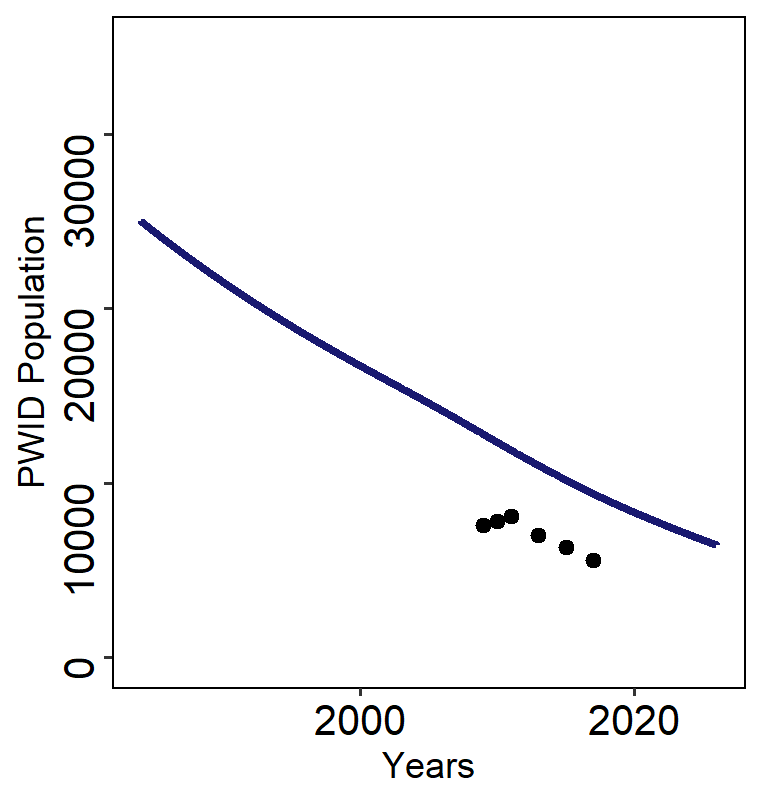** | **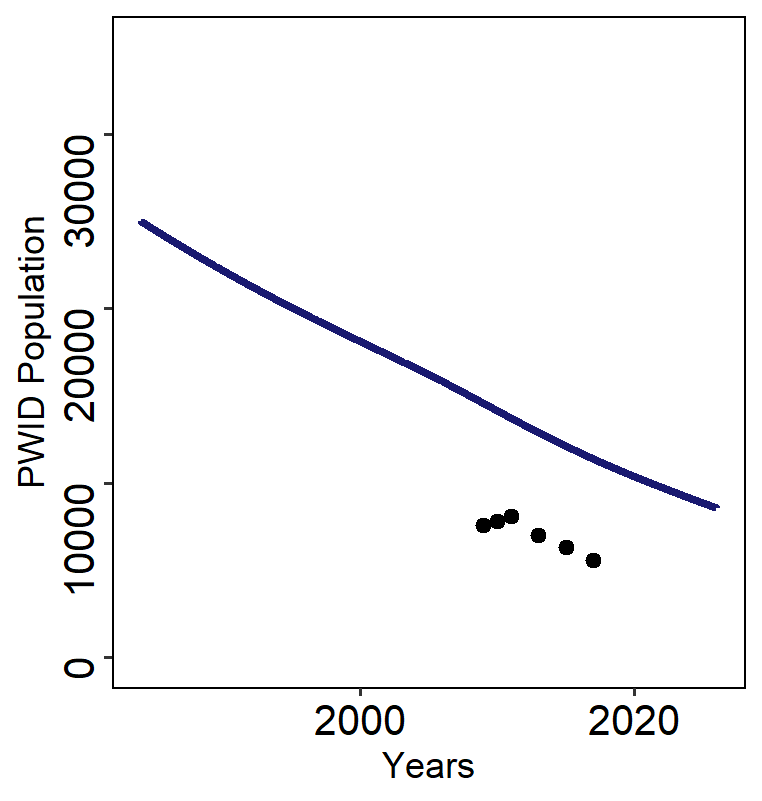** | **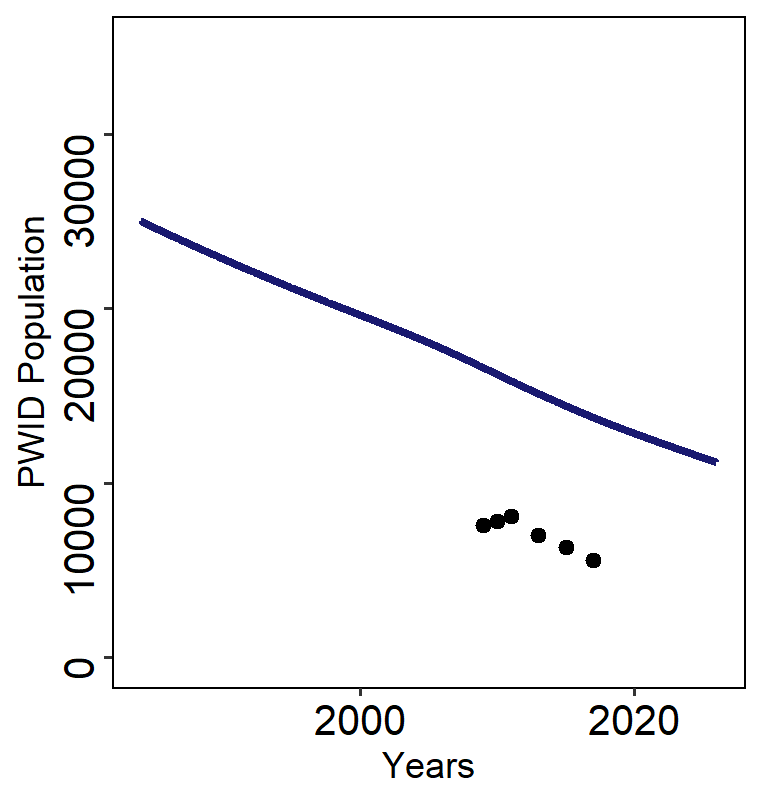** | **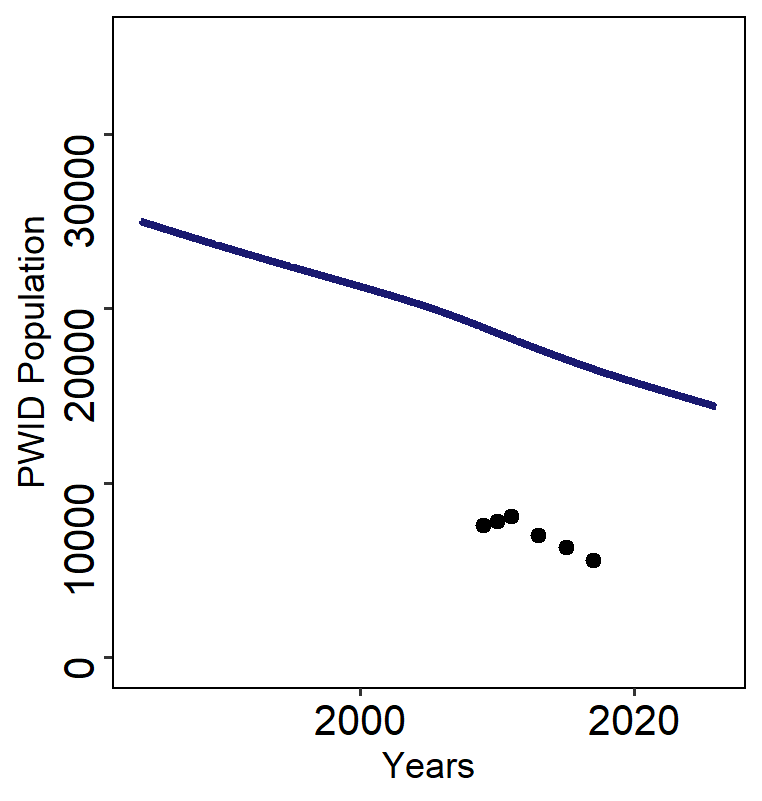** | **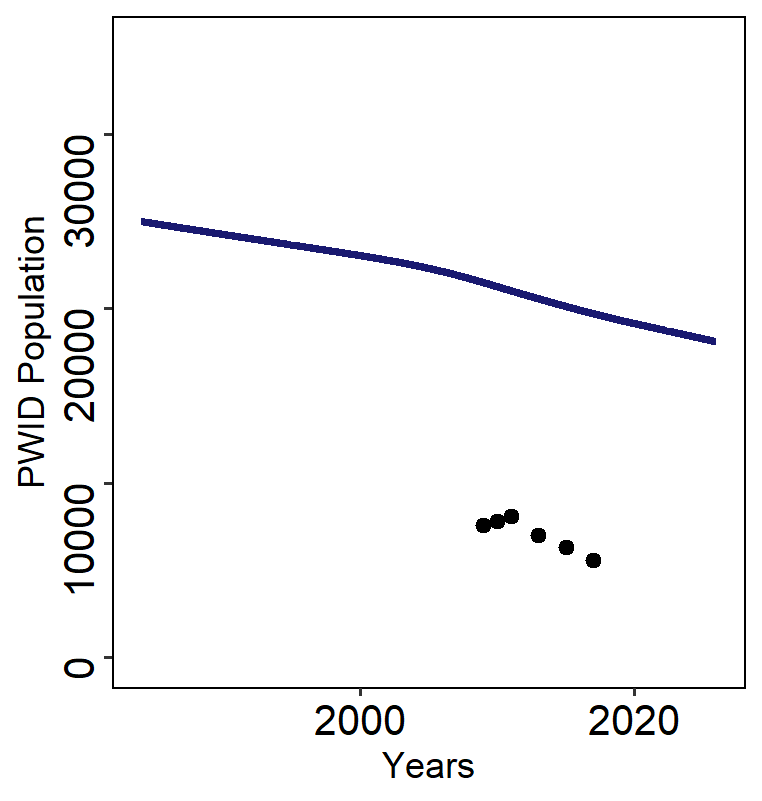** | **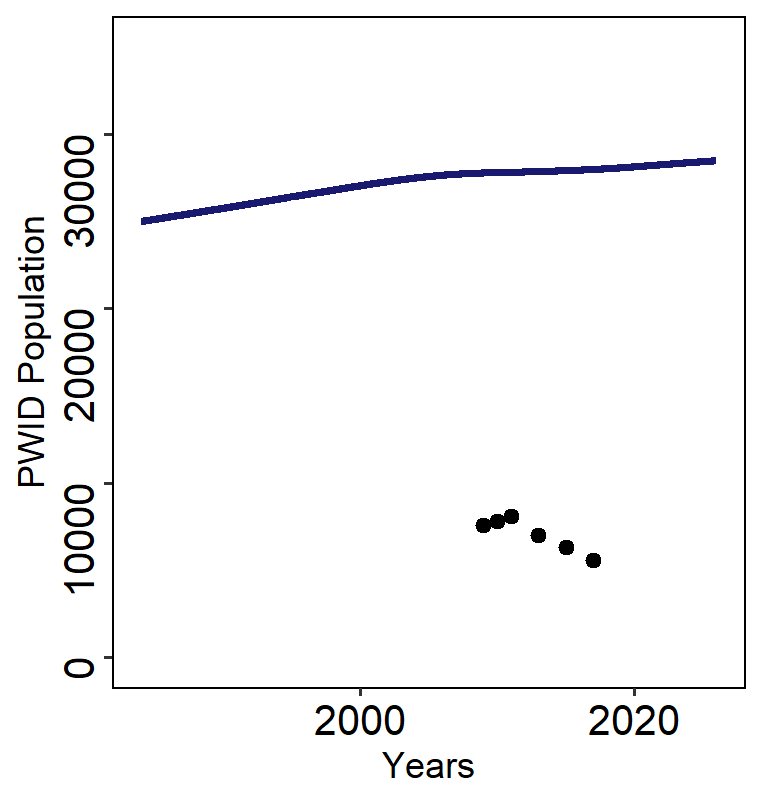** | **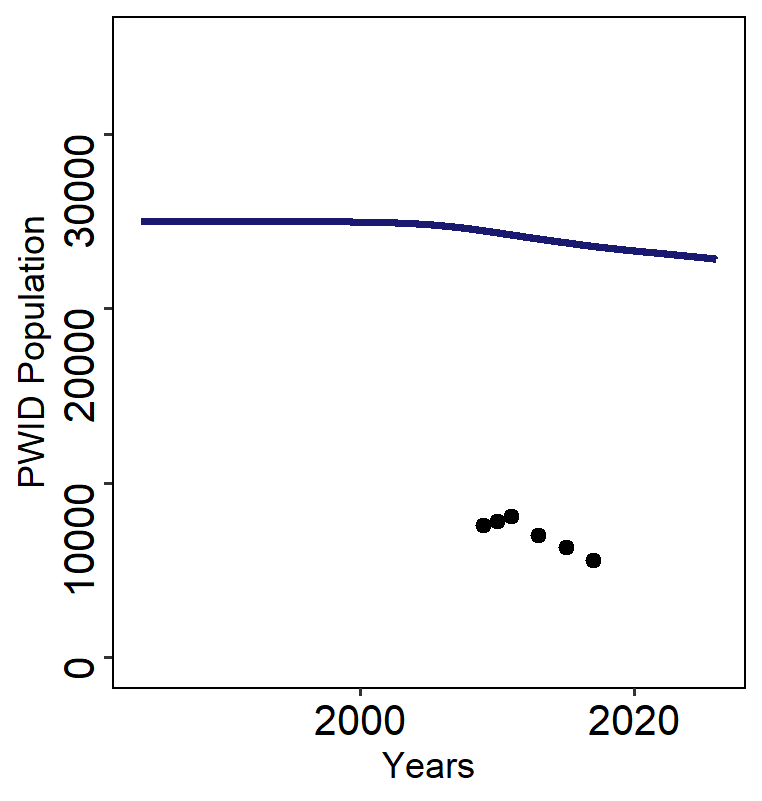** |
|  |  | x-axis: year | | | | | | | | |

**Sensitivity Analysis**

**Fig 3. Sensitivity analysis for max yearly HIV incidence, cumulative true HIV incidence and cumulative mortality at baseline PrEP, OST, NSP, Behavioural interventions coverages.** The y-axis indicates three model outputs: maximum yearly HIV incidence (***imax***), cumulative true HIV incidence (***Cinc_all***) and cumulative mortality (***Cdeath_all***). The x-axis indicates the following input parameters: increasing/decreasing the yearly reference HIV screening coverage by 25% (***screen_scale***), proportion of those who proceed to the 2nd test after receiving positive results on screening (***test2***), proportion of those who proceed to the final confirmatory test (***cnf_test***), population exit rate (***kappa***), population entry rate (***muo***), transmission coefficient (***beta***), transfer from ARTto non-ART status (***omega***)


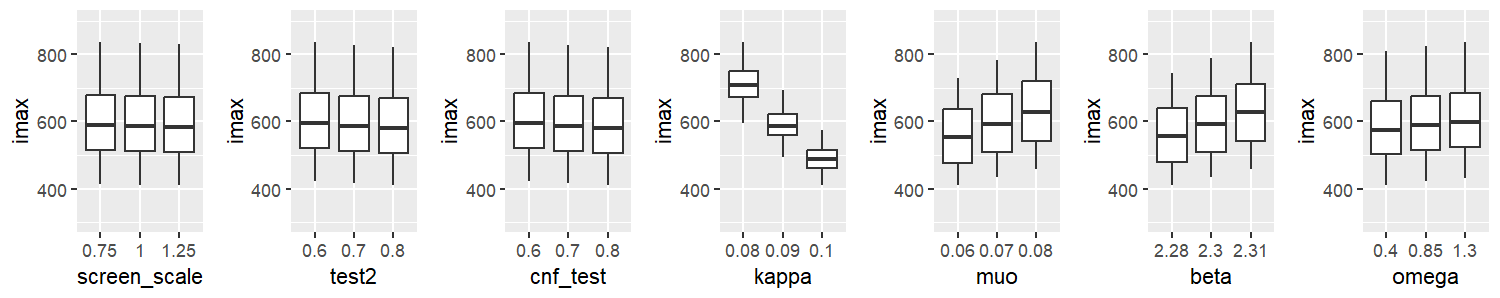


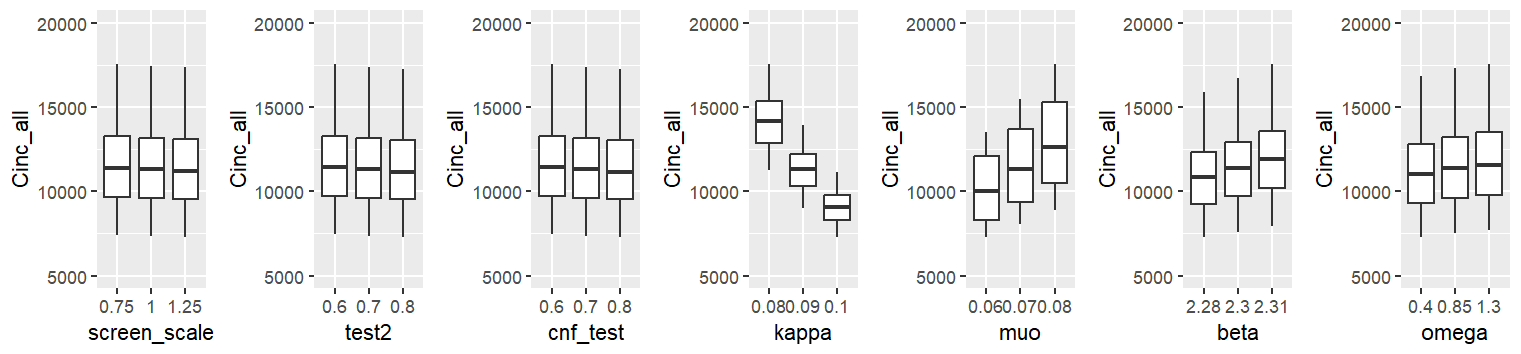


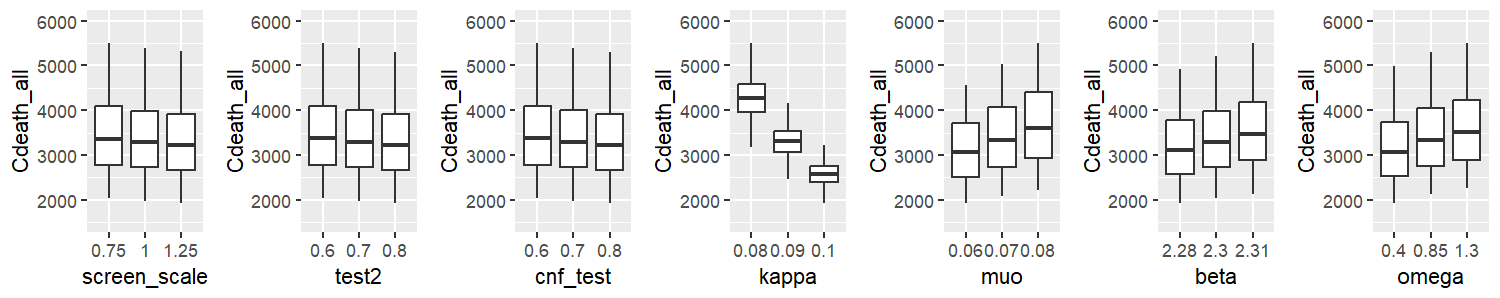


**PWID population structure 2006-2010**

**Fig 4. Distribution of age groups among PWID in Kyrgyzstan.** The bars represent the proportion of each age group from 2006 to 2010. The chart is developed using data from the Kyrgyz HIV Sentinel Surveillance databases for the respective period


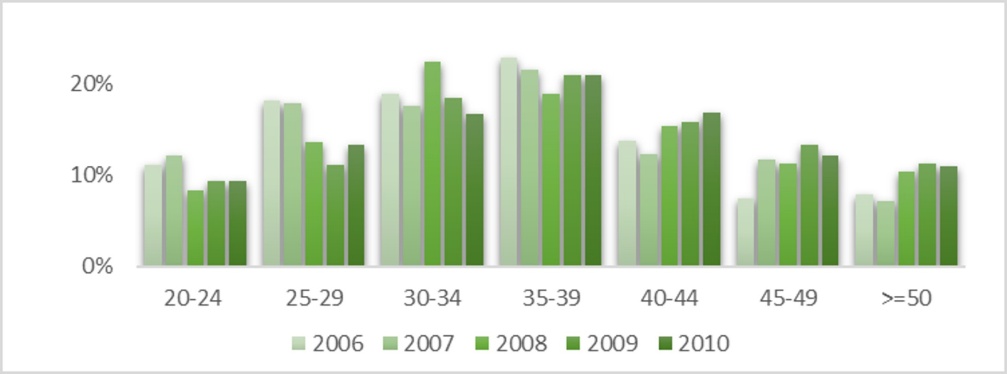


1. WHO. Interim WHO clinical staging of HVI/AIDS and HIV/AIDS case definitions for surveillance: African Region. World Health Organization; 2005. [↑](#footnote-ref-1)
